# Tumor‐derived exosomal miR‐19b‐3p facilitates M2 macrophage polarization and exosomal LINC00273 secretion to promote lung adenocarcinoma metastasis via Hippo pathway

**DOI:** 10.1002/ctm2.478

**Published:** 2021-09-12

**Authors:** Jing Chen, Kai Zhang, Yingru Zhi, Yin Wu, Baoan Chen, Jinyu Bai, Xuerong Wang

**Affiliations:** ^1^ Department of Hematology and Oncology Zhongda Hospital, School of Medicine Southeast University Nanjing Jiangsu P. R. China; ^2^ Department of Respiratory Medicine Nanjing First Hospital Nanjing Medical University Nanjing Jiangsu P. R. China; ^3^ Department of Gastroenterology Nanjing First Hospital Nanjing Medical University Nanjing Jiangsu P. R. China; ^4^ Department of Respiratory Zhongda Hospital Southeast University Nanjing Jiangsu P. R. China; ^5^ Department of Orthopedics The Second Affiliated Hospital of Soochow University Suzhou Jiangsu P. R. China; ^6^ Department of Pharmacology Nanjing Medical University Nanjing Jiangsu P. R. China; ^7^ Center for Clinical Pathology and Laboratory Affiliated Hospital of Yifu Nanjing Medical University Nanjing Jiangsu P. R. China

**Keywords:** exosome, hippo signaling pathway, lung adenocarcinoma, M2 macrophage polarization, miR‐19b‐3p

## Abstract

Numerous reports have elucidated the important participation of exosomes in the communication between tumor cells and other cancer‐related cells including tumor‐associated macrophages (TAMs) in microenvironment. However, the interchange of exosomes between tumor cells and TAMs in the progression of lung adenocarcinoma (LUAD) remains largely enigmatic. Herein, we discovered that LUAD cells induced the M2 polarization of TAMs and the M2‐polarized macrophages facilitated LUAD cell invasion and migration and tumor metastasis in vivo. In detail, LUAD cells secreted exosomes to transport miR‐19b‐3p into TAMs so that miR‐19b‐3p targeted PTPRD and inhibited the PTPRD‐mediated dephosphorylation of STAT3 in TAMs, leading to STAT3 activation and M2 polarization. Also, the activated STAT3 transcriptionally induced LINC00273 in M2 macrophages and exosomal LINC00273 was transferred into LUAD cells. In LUAD cells, LINC00273 recruited NEDD4 to facilitate LATS2 ubiquitination and degradation, so that the Hippo pathway was inactivated and YAP induced the transcription of RBMX. RBMX bound to miR‐19b‐3p to facilitate the packaging of miR‐19b‐3p into LUAD cell‐derived exosomes. Collectively, our results revealed the mechanism underlying the interactive communication between LUAD cells and TAMs through elucidating the exchange of exosomal miR‐19b‐3p and LINC00273 and proved the prometastatic effect of the interchange between two cells. These discoveries opened a new vision for developing LUAD treatment.

## Graphical abstract

LUAD cell‐derived exosomal miR‐19b‐3p induce M2 polarization in THP‐1 cells by targeting PTPRD/STAT3 and STAT3 activated LINC00273 was transmitted by M2 macrophage‐derived exosomes to LUAD cells, activating YAP to induce RBMX‐mediated packaging of miR‐19b‐3p into LUAD cell‐derived exosomes.

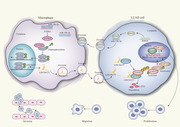

**Abbreviations**
ChIP: chromatin immunoprecipitation
CHX: cycloheximide
Co‐IP: Coimmunoprecipitation
FISH: Fluorescence in situ hybridization
H&E: Hematoxylin and eosin
LATS2: Large tumor suppressor kinase 2
LC: lung cancer
LINC00273: long intergenic non‐protein coding RNA 273
lncRNAs: long non‐coding RNAs
LUAD: lung adenocarcinoma
miRNAs: microRNAs
ncRNAs: Noncoding RNAs
NEDD4: E3 ubiquitin protein ligase NEDD4
NSCLC: Nonsmall cell lung cancer
PTPRD: protein tyrosine phosphatase receptor type D
qRT‐PCR: Quantitative real‐time polymerase chain reaction
RIP: RNA immunoprecipitation
STAT3: Signal transducer and activator of transcription 3
TAMs: Tumor‐associated macrophages
TEM: Transmission electron microscopy
YAP: Yes associated transcriptional regulator

## 1 BACKGROUND

As a leading cause of global cancer death, lung cancer (LC) mainly has two subtypes, small cell lung cancer and nonsmall cell lung cancer (NSCLC).^1^ Lung adenocarcinoma (LUAD) is the most prevalent type of NSCLC, and takes up around half of all LC cases.^2^ However, insufficient screening regimens and atypical early clinical symptoms contributes to a high possibility that patients are diagnosed with advanced or even metastatic LUAD.^3^ Besides, owing to the lack of efficacious therapy, the outcomes of LUAD patients are usually disappointing.^4^ Hence, new biomarkers and therapeutic targets are badly required for improving early detection and refining patient prognosis.

Recently, increasing reports have indicated the correlation of tumor microenvironment with the therapeutic outcomes of various carcinomas.^5^, ^6^ Interaction between tumor cells and other cells in tumor microenvironment is essential for cancer progression.^7^ As is known, tumor microenvironment contains diverse cells and cytokines including tumor‐associated macrophages (TAMs).^8^ Considering the key role of TAMs in tumor development, especially in tumor metastasis,^9^ treating TAMs has been suggested as a new method for cancer treatment.^10^, ^11^ Macrophages are plastic cells that can respond to microenvironmental signals, resulting in high heterogeneity in functions and phenotypes.^12^ Generally, different stimuli trigger two types of macrophage polarization programs so that TAMs can be classified into classical M1 macrophages and alternative M2 macrophages, and these processes have attracted much attention in cancer research.^13^, ^14^ Interestingly, the classically activated M1 macrophages possess proinflammatory, proimmunity, and antitumor functions, whereas M2 macrophages play a cancer‐promoting role by disrupting adaptive immunity and functioning in inflammatory circuits.^15^, ^16^ Importantly, M2 polarization is more likely to happen in the microenvironment of tumors, especially of tumors recurred after treatment.^17^ Besides, the opposite roles of M1 and M2 macrophages in LC have already been reported.^18^ In this work, we focused on the cross‐talking between TAMs and LUAD cells.

Exosomes are regarded as key mediators of inter‐cellular cross‐talk in the tumor microenvironment.^19^ Present literatures demonstrated that tumor‐originated exosomes facilitate angiogenesis, metastasis, and immunosuppression in cancers by altering the phenotype and function of recipient cells.^20^ As an example, exosomes produced by LC cells promote the polarization of macrophage to M2‐like phenotype.^21^ In turn, the exosomes from M2 macrophages have also been proved to accelerate cancer cell migration and invasion.^22^ Herein, we planned to investigate whether exosomes transferred between LUAD cells and macrophages could affect the phenotypes and functions of each other. Exosomes are classified as extracellular vesicles secreted by diverse cell types including tumor cells and TAMs.^23^ There are various cargos wrapped in exosomes, including proteins, lipids, DNA and RNAs, and these molecules are transported to the recipient cells and finally affect their functions.^24^ Of note, exosome‐transmitted non‐coding RNAs (ncRNAs) are vital players in cancer development.^25^ MicroRNAs (miRNAs) and long non‐coding RNAs (lncRNAs), as ncRNAs, in tumor progression have already been unmasked by a great number of studies.^26^, ^27^ However, whether the communication between LUAD cells and macrophages depends on exosomes‐delivered miRNAs or lncRNAs needs to be explored. LINC00273 is recently found to serve oncogenic roles in LC, gastric cancer, and melanoma by promoting metastasis in cancer cells.^28^, ^29^ However, whether LINC00273 participates in the communication between LUAD cells and M2 macrophages is unknown yet. MiR‐19b‐3p is reported as an oncogenic miRNA in several cancers such as cholangiocarcinoma and pancreatic cancer.^30^, ^31^ Interestingly, miR‐19b‐3p also functions as an exosomal miRNA in tubular epithelial cells activating M1 macrophage to affect kidney injury.^32^ Notably, miR‐19b‐3p serves carcinogenic role in NSCLC.^33^ And miR‐19b‐3p mediate the interchange between renal cell cancer cells and cancer stem cells as an exosomal miRNA to promote renal cell cancer.^34^ However, there is not any report about exosomal miR‐19b‐3p in LUAD and its role in the interaction between LUAD cells and M2 macrophages.

Hippo/Yes associated transcriptional regulator (YAP) is an important pathway regulating differentiation, stem cell renewal, and oncogenic transformation.^35^ YAP is the pivotal oncogenic factor in Hippo pathway.^36^, ^37^ Notably, this pathway is substantially reported in LC. For example, Hippo/YAP interacts with FGFR1 so as to sustain stemness of LC cells,^38^ and miR‐135b modulates Hippo pathway to affect LC metastasis.^39^ However, LINC00273 and miR‐19b‐3p have never been related to Hippo/YAP pathway in LC.

In this research, we investigated how the interchange of exosomes affected the functions and phenotypes of LUAD cells and macrophages, and demonstrated the underlying mechanisms mediated by exosomal miR‐19b‐3p and LINC00273. The discoveries might provide new possibilities for the treatment of LUAD, especially for cases with metastasis.

## 2 MATERIALS AND METHODS

### 2.1 Cell lines

A549 (human LUAD cells) and THP1 (human monocytes) were purchased from the American Type Culture Collection (ATCC; Manassas, VA, USA). Human LUAD cells H1975 were bought from the European Collection of Authenticated Cell Cultures (ECACC; Salisbury, UK). For in vitro assays, the culture of THP1, A549 and H1975 cells were conducted in RPMI‐1640 (Gibco, USA) with the addition of 10% fetal bovine serum (FBS, Gibco). For in vivo bioluminescent imaging assay, luciferase‐labeled A549‐Luc2 cells (ATCC) were substituted for A549 cells and cultured in Ham's F‐12K (Kaighn's) Medium (Gibco) supplemented with FBS and 8 μg/mL blasticidin (Sigma‐Aldrich, St. Louis, MO, USA).

**HIGHLIGHTS**

- LUAD cell‐derived exosomal miR‐19b‐3p induced M2 polarization in THP‐1 cells.
- LUAD cell‐derived exosomal miR‐19b‐3p targeted PTPRD/STAT3 in THP‐1 cells, activating LINC00273 transcription.
- M2 macrophage‐derived exosomal LINC00273 was transmitted into LUAD cells to facilitate LUAD metastasis.
- M2 macrophage‐derived exosomal LINC00273 regulated Hippo/YAP pathway by LATS2 ubiquitination, facilitating RBMX‐mediated miR‐19b‐3p packaging into LUAD cell‐derived exosomes.

### 2.2 Macrophage induction from monocytes

THP1 cells cultured in six‐well plates were stimulated with 100 ng/mL phorbol‐12‐myristate‐13‐acetate (PMA; Sigma‐Aldrich) and incubated for 24 to 48 h,^40^ and then maintained in refreshed medium without PMA for 3 days before use.

### 2.3 Culturing medium (CM) treatment

Each small‐molecule inhibitor for our study, including exosome inhibitor GW4869, YAP inhibitor Verteporfin, STAT3 inhibitor NSC 74859, and MST inhibitor XMU‐MP‐1, was provided by MedChemExpress (Monmouth Junction, NJ, USA) and employed for CM in corresponding assays. DMSO (Sigma‐Aldrich) was adopted as the control for inhibitors. A total of 100 U/mL ribonuclease A (RNase A; Thermo Scientific, Waltham, MA, USA) alone or with 1% Triton X‐100 (Invitrogen, Carlsbad, CA, USA) was utilized for detecting exosomal RNA stability by incubating exosomes at 37℃ for 30 min.

### 2.4 Co‐culturing system

To simulate exosome‐mediated intercellular communication between tumor cells and TAMs, an in vitro indirect coculturing system was established with macrophages and LUAD cells (sometimes transfected with Cy3‐labeled RNAs) inoculated, respectively, in the upper and lower chambers of Corning® Transwell® cell culture insert (4 μm pore, Corning Inc., Corning, NY, USA), with a polycarbonate membrane. After 48 h of coculturing, cells were harvested for further assays.

### 2.5 Cell transfection and lentiviral transduction

MiR‐19b‐3p mimics, inhibitors and antagomir‐19b‐3p, as well as negative controls NC mimics, NC inhibitors, and scrambled antagomir (antagomir‐scr), were purchased from RiboBio (Guangzhou, China). The full length of LINC00273, CDS regions of protein tyrosine phosphatase receptor type D (PTPRD), STAT3, YAP, and large tumor suppressor kinase 2 (LATS2), and one single repeat of ubiquitin coding sequence were separately amplified and inserted into pcDNA3.1 (Invitrogen) to construct overexpression plasmids. Short hairpin RNAs (shRNAs) against PTPRD, RBMX, LINC00273, and other lncRNAs, as well as negative control sh‐NC, were purchased from GenePharma (Shanghai, China). Cell transfection was completed using Lipofectamine™ 3000 Transfection Reagent (Invitrogen) according to the manufacturer's protocol. Subsequent assays were performed after 48 h of transfection. Lentiviral transduction was adopted for in vivo assays using Lentiviral Package Kit (Sino Biological, Beijing, China) for 72 h with 8 μg/mL polybrene (Sigma‐Aldrich) to improve transduction efficiency. The sequences of oligonucleotides for cell transfection are presented in Supporting information Table S1.

### 2.6 Exosome isolation and purification

Exosomes were isolated from CM by means of sequential ultracentrifugation (Beckman Coulter, Indianapolis, IN, USA). In brief, CM for the assay was substituted with RPMI‐1640 with 10% exosome‐depleted FBS (undergoing 100 000 × *g* ultracentrifugation for 8 h). After 72 h of cell culturing, CM was collected by centrifugation (300 × *g*) for 10 min. Subsequently, residual cells and debris were removed through 2000 × *g* centrifugation for 10 min and 10 000 × *g* centrifugation for 30 min. Then exosomes were extracted from CM by 100 000 × *g* ultracentrifugation for 70 min. Then, supernatant was discarded, while the pellets were washed, ultracentrifuged, and resuspended in phosphate‐buffered saline (PBS). Each process of ultracentrifugation was performed at 4℃. For exosome treatment, the CM of recipient cells was supplemented with purified exosomes at 1 μg/mL unless otherwise specified.

### 2.7 Transmission electron microscopy (TEM)

Exosome sample for transmission electron microscopy (TEM) analysis was suspended in 2.5% glutaraldehyde for 2 h and rinsed with PBS. Then, 20 μL of exosome suspension was dropped on a small carbon‐coated copper grid (Electron Microscopy Sciences, Hatfield, PA, USA), and excess suspension was removed with filter paper. A total of 3% phosphotungstic acid was applied to the grid for exosome staining for 1 min. After drying, the morphology of stained exosomes was observed using TEM Tecnai G2 F20 (FEI company, Oregon, USA).

### 2.8 Fluorescence labeling and tracing of exosomes or exosomal RNAs

The process of exosomes or exosomal RNAs transmitted to recipient cells was illustrated by fluorescence microscopy. Vybrant™ DiO Cell‐Labeling Solution (Invitrogen) was applied for exosome labeling. A total of 1 mL exosome suspension was supplemented with 5 μL 3,3′‐dioctadecyloxacarbocyanine perchlorate (DiO; green) solution following incubation at 37℃ for 20 min. After three repeats of centrifugation, washing, and 10 min of recovery, DiO‐labeled exosomes were added to the CM of recipient cells. Biosynthesized miR‐19b‐3p or LINC00273 (GenePharma) was labeled with cyanine3 (Cy3; red) using Arcturus® Turbo Labeling™ Kit of Cy3 Dye (Applied Biosystems, Foster City, CA, USA). Then cells containing Cy3‐labeled RNAs were subjected to coculturing system for evaluating the process of exosomal RNAs transported to recipient cells. After 0, 1, 2, 4, 6, 12, 24, or 48 h of exosome treatment or coculturing, cells for fluorescence labeling underwent 4% paraformaldehyde (Sigma‐Aldrich) fixation for 5 min and underwent permeabilization with 0.01% Triton X‐100 (Invitrogen) for 10 min. Subsequently, phalloidin conjugated with tetramethylrhodamine isothiocyanate (TRITC; red; R&D Systems) or fluorescein isothiocyanate (FITC; green; R&D Systems) was adopted for labeling cytoskeleton at a concentration of 150 nM for 20 min. Besides, 4’,6‐diamidino‐2‐phenylindole (DAPI; Abcam, Cambridge, MA, USA) was applied for nuclear staining. Leica DMi8 inverted microscope (Leica Microsystems, Wetzlar, Germany) with LAX software was used for observing exosome uptake images, and the percentage of DiO‐positive or Cy3‐positive cells was measured.

### 2.9 Animal model

Animal experiments were approved by the Nanjing First Hospital, Nanjing Medical University. Four‐week‐old male BALB/c immunodeficient mice provided by the Vital River Laboratory Animal Technology Co., Ltd. (Beijing, China) were preserved in a pathogen‐free animal facility under standard breeding conditions (24℃ constant room temperature, 70% humidity, 12 h/12 h controlled light/dark cycle and supply of standard food and water). Luciferase‐labeled A549‐Luc2 cells (2 × 10^6^) and PMA‐induced THP1 cells (2 × 10^6^) were resuspended in 200 μL PBS for injection into mice in mixture.^41^ Mice were randomly divided into different groups, which were respectively subjected to tail vein injection (n = 30, 5 mice/group) or femoral cavity injection (n = 24, 4 mice/group) with the suspended mixture of cocultured cells. For osseous metastasis by femoral cavity injection, mice were anesthetized by 3% isoflurane, and the superficial incision (0.5 to 1 cm) was created near the knee so as to expose patellar ligament. Thereafter, the femoral cavity of each mouse was inserted with a needle (25‐gaugeat) at a site of the intercondylar notch on left femur, and the inserted needle was substituted with a microinjection syringe (10 μL) containing mixed cells added with absorbable gelatin sponge solution that facilitates injection site closure. Then, the femoral cavity was slowly injected with contents in syringe for 2 min. Silicone adhesive (Kwik‐Sil, World Precision Instruments) was used to seal the outside site of injection to prevent cells from leaking out of articular cavity. Mice in tail vein or femoral cavity injection group were subjected to in vivo bioluminescent imaging assay before euthanasia and metastasis tissue staining.

### 2.10 In vivo bioluminescent imaging assay

Mice for the assay were anesthetized and intraperitoneally injected with d‐luciferin potassium salt (30 mg/kg body weight; Sigma‐Aldrich). After 10 min, mice were placed on IVIS Lumina III In Vivo Imaging System (PerkinElmer, Waltham, MA, USA) on ventrodorsal position. Bioluminescent images illustrating hepatic and pulmonary metastasis for tail vein injection group, or osseous metastasis for femoral cavity injection group were recorded with a 5‐min exposure period.

### 2.11 Hematoxylin and eosin (H&E) staining

After metastasis examination by bioluminescent imaging assay, mice were euthanized and anatomized to obtain tissues from putative metastasis locations. Tissue samples were fixed by paraformaldehyde for 1 day and paraffin embedded. For osseous samples, tissues also underwent decalcification and dissection before paraffin treatment. Rotary Microtome (Leica, Frankfurt, Germany) was then utilized for tissue sample sectioning into pieces (5‐10 μm in thickness). After xylene‐deparaffinizing at 37°C for 20 min, sections were stained using Hematoxylin and eosin (H&E) Staining Kit (Sangon Biotech, Shanghai, China). BX53 Upright Microscope (Olympus, Tokyo, Japan) was used for morphological observation.

### 2.12 Quantitative real‐time polymerase chain reaction (qRT‐PCR)

RNA expression was detected through qRT‐PCR. To isolate total RNA, TRIzol™ Reagent (Invitrogen) was adopted, while ExoQuick® Exosome RNA Column Purification Kit (System Biosciences, Palo Alto, CA, USA) was adopted for exosomal RNA extraction. DNase I, Amplification Grade (Invitrogen) was applied to digest DNA contaminants. Nanodrop™ 2000 spectrophotometer (Thermo Scientific) was used to detect the concentration and quality of RNA samples. RevertAid First Strand cDNA Synthesis Kit (Thermo Scientific) (for mRNA and lncRNA) and TaqMan MicroRNA Assays (Applied Biosystems, Waltham, MA) (for miRNA) was applied for synthesizing complementary DNAs (cDNAs) from 1 μg of RNA samples. qRT‐PCR was performed on CFX96 Touch Real‐Time PCR Detection System (Bio‐Rad, Hercules, CA, USA) using SYBR™ Green PCR Master Mix (Applied Biosystems). All data were normalized to the internal control GAPDH or U6, and 2^–ΔΔ^ *^Ct^* method was used for relative expression quantification. The sequences of qRT‐PCR primers (RiboBio) are presented in Supporting information Table S2.

### 2.13 Western blot analysis

Cellular or exosomal protein samples were obtained using Radioimmunoprecipitation assay (RIPA) Lysis and Extraction Buffer (Thermo Scientific) supplemented with Halt™ Protease and Phosphatase Inhibitor Cocktail (Thermo Scientific). Cytoplasmic‐Nuclear RNA Purification Kit (Norgen Biotek, Thorold, ON, Canada) was utilized for subcellular fractionation before lysing if necessary. Lysates were quantified using Bradford reagent (Bio‐Rad), and 30 μg protein sample was loaded onto 10% sodium dodecyl sulfate‐polyacrylamide gel electrophoresis (SDS‐PAGE) gel. The electrophoresis separated proteins were transferred onto polyvinylidene difluoride (PVDF) membranes (Millipore, Billerica, MA, USA), which were then blocked with Membrane Blocking Solution (Invitrogen) and incubated with specific primary antibodies from Abcam (Cambridge, UK), or Cell signaling technology (Danvers, MA, USA), at 4℃ overnight. Then secondary antibody (Abcam) was applied at 37℃ for 1 h, and the membranes were visualized using Amersham™ enhanced chemiluminescence (ECL) Plex™ system (Amersham Pharmacia, Piscataway, NJ, USA).

### 2.14 Flow cytometry

To detect macrophage surface markers, cells resuspended in cold PBS were incubated with anti‐human CD206‐APC or with anti‐human CD206‐APC plus anti‐human CD11b‐FITC (Invitrogen) at 4℃ for 30 min. After washing, labeled cells were subjected to flow cytometry using BD Accuri™ C6 flow cytometer (BD Biosciences, San Jose, CA, USA) to detect the ratio of CD206‐positive or CD206 plus CD11b‐positive macrophages.

### 2.15 Cell counting kit 8 (CCK‐8)

LUAD cells were suspended and cultured in the medium (100 μl) in the 96‐well plate. The cells were cultured for 24, 48, and 72 h. Then, the medium was added with CCK‐8 reagent (10 μL, Sangon Biotech Co., Ltd.) and cells were subjected to incubation for 4 h at 37℃. Detection of optical density (OD) levels (450 nm) was finished by a microplate reader.

### 2.16 Wound healing assay

Wound healing assay was conducted when cells seeded in six‐well plates reached 90% confluence. A scratch across cell monolayer was generated by A sterile pipette tip to form a linear wound, and cell debris were washed away with PBS. After 24 h of incubation, wound healing was visualized using a microscope (Olympus), and the relative width of wound healing was measured.

### 2.17 Transwell assay

Cells in upper chamber of Matrigel‐coated Corning® Transwell® cell culture insert (Corning Inc.) with an 8.0 μm pore polycarbonate membrane were cultured in conditioned medium or medium with exosome treatment. Meanwhile, complete medium containing FBS was added in the lower chamber. After 48 h of incubation, cells invading to lower chamber fixed by paraformaldehyde were stained with 0.1% crystal violet. Invaded cell number was counted with the help of a microscope (Olympus).

### 2.18 Luciferase reporter assay

To illustrate the molecular interaction between PTPRD mRNA and miR‐19b‐3p, pmirGLO Dual‐Luciferase miRNA Target Expression Vectors (Promega) were adopted. The 3′‐untranslated region (UTR) sequence of PTPRD mRNA was inserted into pmirGLO vector, constituting wild‐type (WT) luciferase reporter (PTPRD‐WT). Meanwhile, mutant (Mut) PTPRD 3′‐UTR sequence, which contained single or double mutant putative miR‐19b‐3p binding site(s) established by QuikChange Site‐Directed Mutagenesis Kit (Stratagene, La Jolla, CA, USA), was used for constructing mutant luciferase reporter (PTPRD‐Mut1, Mut2, or Mut1&2). Macrophages were transfected with empty vectors, WT reporters or mutant reporters, as well as NC mimics or miR‐19b‐3p mimics.

To evaluate the level of YAP signaling activity, 8x GTIIC‐luciferase vectors were adopted. For different assays, A549 or H1975 cells were transfected with 8x GTIIC‐luciferase vectors and pRL Renilla Luciferase Control Vectors (Promega) for normalization, together with sh‐NC or various shRNAs, pcDNA3.1, or pcDNA3.1/LINC00273, or exosome treatment.

To detect gene transcriptional activity, pGL3 Luciferase Reporter Vectors (Promega) were adopted. WT LINC00273 or RBMX promoter sequence (LINC00273 or RBMX‐Pro‐WT), mutant sequence with single or double mutant putative STAT3 or TEAD4 binding site(s) (LINC00273 or RBMX‐Pro‐Mut1, Mut2 or Mut1&2), or several promoter sequence truncations, were respectively cloned into pGL3 vectors to construct corresponding luciferase reporters. Cells were transfected with empty vectors or luciferase reporters together with pRL Renilla Luciferase Control Vectors, and treated with NSC 74859 (for LINC00273‐Pro reporters in macrophages) or Verteporfin (for RBMX‐Pro reporters in A549 or H1975 cells) if necessary. Finally, relative luciferase activity (firefly/Renilla luciferase activity ratio) for each group was measured using Dual‐Luciferase® Reporter Assay System (Promega).

### 2.19 RNA immunoprecipitation (RIP) assay

EZ‐Magna RIP™ RNA‐Binding Protein Immunoprecipitation Kit (Millipore) was applied for RIP. A549 or H1975 cells for the assay underwent transfection of pcDNA3.1 or pcDNA3.1/LINC00273 beforehand. After rinsing with PBS, cells or exosomes were incubated in RIP lysis buffer supplemented with protease inhibitors and RNase inhibitors on ice for 5 min to obtain lysates. Next, 100 μL of lysates were subjected to immunoprecipitation using magnetic beads conjugated with antibody against NEDD4, SNRNP70, RBMX or the negative control immunoglobulin G (IgG) in RIP buffer. After overnight incubation at 4℃, immunoprecipitated RNAs were digested by proteinase K, and the level of LINC00273, U1, or miR‐19b‐3p for each assay was measured by qRT‐PCR.

### 2.20 RNA pull‐down assay

LINC00273 and its antisense sequence, as well as miR‐19b‐3p and its mutant sequence with mutant RBMX binding motif (CCAU to GGUA), were constructed in vitro by GenePharma. Biotin RNA Labeling Mix (Roche, Basel, Switzerland) was then applied to construct biotinylated probes (Bio‐LINC00273‐Sense or Antisense, and Bio‐miR‐19b‐3p‐WT or Mut). To conduct pull‐down assay, in brief, biotinylated probes and Pierce™ streptavidin agarose beads (Thermo Scientific) were mixed and cultivated at 4℃ overnight. Then cell or exosome lysates and RNase inhibitor were added. After 1 h of incubation on ice, the eluted proteins or RNAs were analyzed by mass spectrometry, qRT‐PCR, or western blot.

### 2.21 Mass spectrometry

Through RNA pull‐down assay using Bio‐LINC00273‐Sense or Antisense probes, the proteins from A549 cells were subjected to SDS‐PAGE, followed by silver staining with the help of ProteoSilver™ Silver Stain Kit (Sigma‐Aldrich). The protein bands in Bio‐LINC00273‐Sense group in comparison with Bio‐LINC00273‐Antisense group were excised and analyzed by Q Exactive mass spectrometer (Thermo Scientific).

### 2.22 Coimmunoprecipitation (Co‐IP) assay

Coimmunoprecipitation (Co‐IP) assay was utilized for detecting LATS2 ubiquitination level or evaluating the binding between LATS2 and NEDD4. For ubiquitination assay, A549 or H1975 cells for the assay were additionally transfected with the overexpression plasmids for ubiquitin and LATS2 in order to guarantee co‐IP efficiency. After plasmid transfection or exosome treatment, cells were treated with 10 nM proteasome inhibitor MG‐132 (MedChemExpress) or RPMI‐1640 and cultivated for additional 8 h. After Pierce™ IP Lysis Buffer (Thermo Scientific) treatment on ice for 5 min, 100 μL of cell lysates underwent immunoprecipitation with LATS2 antibody (Proteintech, Chicago, IL, USA) on Pierce Protein A/G Magnetic Beads (Life Technologies, Carlsbad, CA, USA) with rotation at 4℃ overnight and treated with elution buffer (Thermo Scientific). Ubiquitination level was evaluated by western blot using ubiquitin antibody (Abcam). To detect LATS2‐NEDD4 interaction, cell lysates were cocultured with anti‐NEDD4 (Proteintech) or IgG (Abcam) conjugated magnetic beads, and the eluted proteins underwent western blot with antibody against LATS2 or NEDD4.

### 2.23 Chromatin immunoprecipitation (ChIP) assay

EZ‐Magna ChIP™ A/G Chromatin Immunoprecipitation Kit (Millipore) was employed for this experiment. In brief, cells (after LINC00273 overexpression or inhibitor treatment if necessary) were subjected to 4% formaldehyde treatment at 37℃ for 10 min for chromatin protein‐DNA crosslinking. Then crosslinked chromatin was extracted, resuspended in lysis buffer and sonicated with Q700 Sonicator (Qsonica, Newtown, CT, USA) into small fragments (500‐1000 bp). Next, for immunoprecipitation, chromatin fragments were cocultured with antibody against STAT3 (Invitrogen), TEAD1 (LSBio), TEAD4 (Abcam), YAP (Invitrogen), or IgG (Abcam) conjugated on magnetic beads at 4℃ overnight. After washing and DNA purification, the quantification of immunoprecipitated LINC00273 promoter (for macrophages) or RBMX promoter (for A549 or H1975 cells) was conducted by quantitative PCR.

### 2.24 Fluorescence in situ hybridization (FISH) and immunofluorescence (IF) assay

For Fluorescence in situ hybridization (FISH) labeling, LINC00273 probe (GenePharma) was fluorescence‐labeled using FISH Tag™ RNA Red Kit with Alexa Fluor® 594 dye (red; Invitrogen). A549 cells, which had been transfected with pcDNA3.1/LINC00273, were incubated with denatured probe (at 80℃ for 2 min) in hybridization buffer at 55℃ overnight. Next, for immunofluorescence labeling, cells were rinsed with PBS, immobilized with paraformaldehyde, permeabilized with Triton X‐100 and blocked with 1% bovine serum albumin (BSA; Sigma‐Aldrich) for 30 min. Subsequently, cells were incubated with NEDD4 antibody (Invitrogen), 1% BSA, and 0.1% Tween 20 (Sigma‐Aldrich) in PBS at 4℃ overnight. Alexa Fluor® 488‐conjugated goat polyclonal anti‐rabbit IgG (green; Abcam) secondary antibody was then applied at 37℃ for 1 h of incubation. DAPI was used for nuclear staining. Cells were observed using a Leica DMi8 inverted microscope (Leica Microsystems).

### 2.25 Cycloheximide (CHX) chase assay

A549 or H1975 cells for the assay were treated with 10 μg/mL protein synthesis inhibitor cycloheximide (CHX) (MedChemExpress) and cultivated for 0, 6, or 12 h. Then cell lysates were subjected to western blot to detect the remaining level of LATS2 protein.

### 2.26 Bioinformatics analysis

The expression profiles of miRNAs or lncRNAs between blood samples from cancer patients and healthy individuals were obtained from BBCancer database (http://bbcancer.renlab.org/) and illustrated using an MA plot. ENCORI database (http://starbase.sysu.edu.cn/) was employed to predict miRNAs targeting the indicated gene. UCSC Genome Browser (https://genome.ucsc.edu/) and JASPAR database (http://jaspar.genereg.net/) were applied to predict potential binding sites for specific transcription factors. The putative RNA‐binding protein (RBP) for miR‐19b‐3p was predicted by RBPDB (http://rbpdb.ccbr.utoronto.ca/).

### 2.27 Statistical analysis

Data analysis was performed on SPSS 22.0 software (SPSS Inc., Chicago, IL, USA). Each assay had at least three independently repeats. Quantitative results were presented as mean ± standard deviation (SD). Student's *t*‐test or one‐way analysis of variance (ANOVA) was respectively applied to analyze statistical significances of two groups or multiple groups. *P* < 0.05 suggested statistically significance.

## 3 RESULTS

### 3.1 LUAD cells induce M2 polarization of macrophages to promote LUAD tumor metastasis

To explore the role of LUAD cell‐induced M2 polarization in LUAD tumor metastasis, we cocultured A549 cells and PMA‐induced THP1 cells (named as THP1 (Mφ) in subsequence) in a transwell system in vitro. Independently PMA‐induced THP1 cells were separately used as the negative controls. First, through in vitro flow cytometry analysis, we determined that the positive expression rate of macrophage marker (CD11b) and M2 macrophage‐related phenotypic marker (CD206) was increased in macrophages in coculture group compared with the independent culture group (Supporting information Figure S1A), indicating that LUAD cells activated M2 polarization of macrophages. Subsequently, LUAD cells and M2 macrophages were mixed and injected in mice in different ways to establish animal model (Supporting information Figure S1B). To explore the role of the cells above in hepatic metastasis and pulmonary metastasis, we injected the cells into the nude mice through tail vein. The bioluminescent imaging reveals that coinjection of A549 and THP1 (Mφ) cells aggravated hepatic and pulmonary metastasis in mice (Supporting information Figure S1C). Through H&E staining, we observed the increase of hepatic and pulmonary metastatic nodules in mice coinjected A549 and THP1 (Mφ) cells (Supporting information Figure S1D). Furthermore, osseous metastatic model was established through femoral cavity injection and we observed that the injection of mixed A549 and THP1 (Mφ) cells facilitated osseous metastasis (Supporting information Figure S1E). These data indicated that THP1 (Mφ) cells participated in LUAD metastasis in vivo, so we hypothesized that in LUAD microenvironment, THP1 (Mφ) cells may also be stimulated to polarize into M2 phenotype by LUAD cells so as to further facilitate tumor metastasis.

**FIGURE 1:**
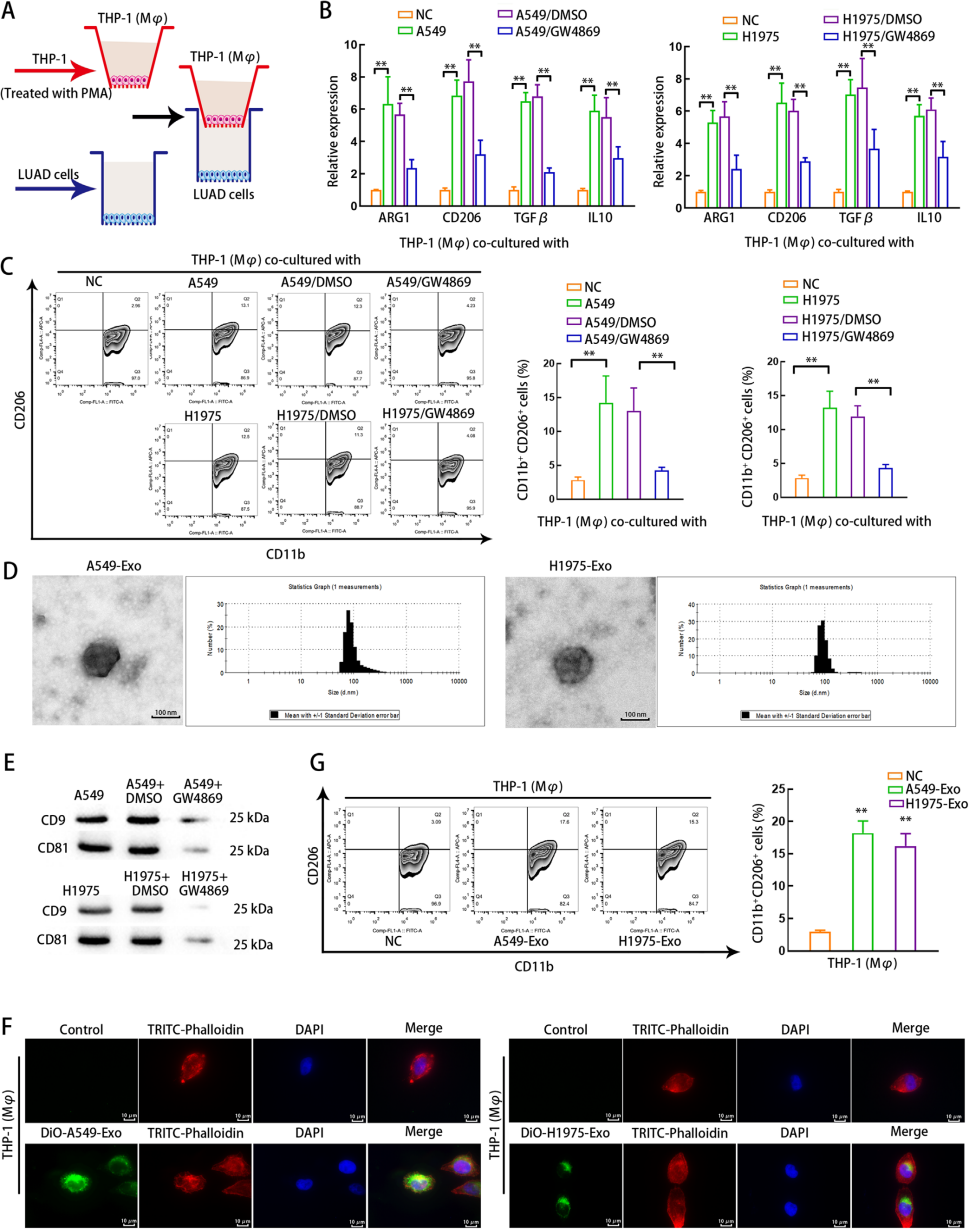
LUAD cells induces M2 macrophage polarization by secreting exosomes. (A) Schematic illustration of the co‐culturing model for macrophages and LUAD cells using a transwell chamber. After PMA induction, THP1 (Mφ) were mono‐cultured (NC) or co‐cultured with untreated LUAD cells (A549 or H1975), LUAD cells treated with DMSO (A549/DMSO or H1975/DMSO), or LUAD cells treated with 5 μM of exosome secretion inhibitor GW4869 (A549/GW4869 or H1975/GW4869). (B) qRT‐PCR was adopted to detect the levels of M2 markers (ARG1, CD206, TGFβ and IL10) in THP1 (Mφ) in each group of co‐culturing system. (C) Flow cytometry was applied to measure macrophage marker (CD11b) and M2 macrophage‐related phenotypic marker (CD206) on the surfaces of macrophages in each group. The ratio of CD206‐positive plus CD11b‐positive (CD206+CD11b+) macrophages of each group were quantitated. (D) TEM images and NTA analysis results of A549‐Exo or H1975‐Exo. Scale bar: 100 nm. (E) Western blot of exosome markers CD9 and CD81 in extracted exosomes from LUAD cells treated without or with DMSO or GW4869. (F) Fluorescence microscopy images illustrated the process of LUAD cell‐derived exosomes transmitted to macrophages. A549 and H1975 cell‐derived exosomes were labeled by DiO (green). TRITC‐Phalloidin (red) was adopted for labeling cytoskeleton of THP1 (Mφ) cells. Scale bar: 10μm. (G) Flow cytometry was applied to measure CD206+CD11+ macrophages in each group without (NC) or with LUAD cell‐derived exosome treatment. THP1 (Mφ), macrophages induced from THP1 cells. A549‐Exo or H1975‐Exo, exosomes derived from A549 or H1975 cells. Data are presented as mean ± SD of at least three independent experiments. **p < 0.01

Therefore, we started with exploring how LUAD cells mediated the M2 polarization of macrophages. At first, LUAD cells and THP1 (Mφ) cells were incubated in a transwell coculture system (Figure 1A). Two days later, we detected the levels of M2 macrophage‐related phenotypic markers in four different groups, with the independently cultured THP1 (Mφ) cells as control group. In Figure 1B, the levels of M2 markers were overtly increased in THP1 (Mφ) cells of A549/H1975 coculture group, and treatment of GW4869 (exosome inhibitor) reversed the increase in levels of M2 markers in THP1 (Mφ) cells cocultured with LUAD cells. However, we found that levels of M1 markers, including TNF‐α, NOS2, CXCL9, and CXCL10, were not changed in groups mentioned above (Supporting information Figure S9A). Next, flow cytometry analysis was conducted to examine the positive expression of CD206 and CD11b. Consistently, an increased ratio of CD206+CD11b+ macrophages was achieved in A549/H1975 coculturing group, but such increase was reversed under GW4869 treatment (Figure 1C). These data suggested that LUAD cells induce M2 polarization of macrophages in an exosome‐dependent manner. Accordingly, we monitored the exosomes derived from LUAD cells using TEM, and nanoparticle tracking analysis (NTA) confirmed that the exosomes we extracted were around 100 nm in diameter (Figure 1D). Western blot validated that exosome markers CD9 and CD81 were detected in exosomes from LUAD cells treated without or with DSMO, and the markers above diminished after GW4869 treatment (Figure 1E). Next, the LUAD‐derived exosomes labeled with DiO were collected and were used to treat THP1 (Mφ) cells. We observed that THP1 (Mφ) cells showed DiO positivity at 24 h after treatments with LUAD cell‐derived exosomes, but THP1 (Mφ) cells without LUAD exosome treatment did not show DiO fluorescence (Figure 1F). Moreover, we validated that THP1 (Mφ) cells showed higher percentage of CD11b+CD206+ population after being cultured with exosomes derived from A549 and H1975 cells (Figure 1G). These data underlined that LUAD cells induce M2 polarization of macrophages in an exosome‐dependent manner.

### 3.2 Exosomes derived from LUAD cells regulate PTPRD/STAT3 axis through transmitting miR‐19b‐3p

Exosomes are known as important intercellular mediators which can transmit RNA molecules from donor cells to recipient cells. Here, we detected whether LUAD cell‐derived exosomes regulated M2 polarization through transmitting a certain RNA molecule. First, the levels of key factors of pathways involved in M2 polarization were detected.^42^, ^43^, ^44^ Consequently, only p‐STAT3 level was significantly enhanced in THP1 (Mφ) cells treated with A549‐Exo or H1975‐Exo (Supporting information Figure S2A). Subsequently, we examined the level of enzymes regulating STAT3 phosphorylation.^45^, ^46^, ^47^, ^48^, ^49^, ^50^, ^51^, ^52^, ^53^, ^54^, ^55^, ^56^, ^57^, ^58^, ^59^ Among all these enzymes, only PTPRD presented significant downregulation in THP1 (Mφ) cells under A549‐Exo or H1975‐Exo treatment (Supporting information Figure S2B). Meanwhile, we observed the decline of PTPRD protein level in THP1 (Mφ) cells cultured with LUAD cell‐derived exosomes (Supporting information Figure S2C). Subsequently, we found that PTPRD level was downregulated and p‐STAT3 level was upregulated in THP1 (Mφ) cells in LUAD cell coculture group, and the results were reversed in THP1 (Mφ) cells when the cocultured LUAD cells were treated with GW4869, and STAT3 level was not changed all the way (Supporting information Figure S2D). Further, we investigated whether PTPRD downregulation was attributed to certain exosomal miRNAs from LUAD cells. Based on the miRNA expression profile in LUAD samples obtained from BBCancer database, MA Plot was generated (miRNAs were defined to be upregulated when Log2FC > 1). We chose the top 10 upregulated miRNAs possessing binding sites with PTPRD for subsequent analysis (Supporting information Figure S2E). Next, the 10 candidate miRNAs were subjected to qRT‐PCR analysis in THP1 (Mφ) cells treated with or without A549/H1975‐Exo. Of all these miRNAs, miR‐19b‐3p was significantly upregulated in THP1 (Mφ) cells treated with A549‐Exo or H1975‐Exo (Supporting information Figure S2F). Importantly, miR‐19b‐3p level in the culture medium of A549 and H1975 cells was not degraded under RNase A treatment. Nevertheless, miR‐19b‐3p degradation was effectively triggered by RNase A after cotreatment with Triton X‐100 (Supporting information Figure S2G), indicating that extracellular miR‐19b‐3p was protected by membranes rather than directly exposed outside. Later, we validated that miR‐19b‐3p level was higher in LUAD cells than in THP1 (Mφ) cells (Supporting information Figure S7A). Exosomal miR‐19b‐3p was expressed higher than miR‐19b‐3p expression in LUAD cells (Supporting information Figure S7B), indicating the enrichment of miR‐19b‐3p in LUAD exosomes. Taken together, miR‐19b‐3p might work as an exosomal miRNA from LUAD cells to regulate PTPRD/STAT3 axis in THP1 (Mφ) cells.

### 3.3 Silencing of miR‐19b‐3p in LUAD cells reverses the M2 polarization in THP1 (Mφ) cells

**FIGURE 2:**
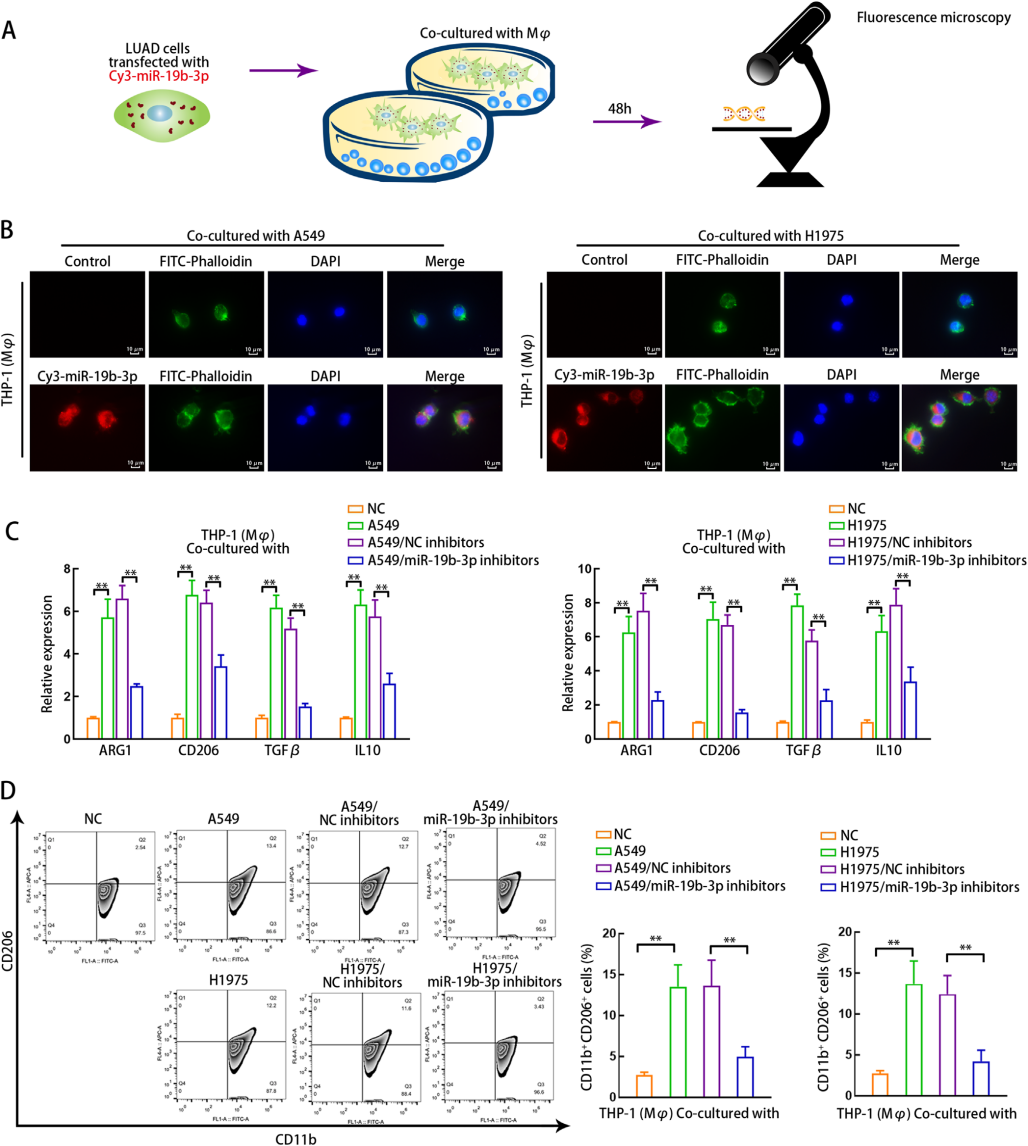
miR‐19b‐3p knockdown in LUAD cells inhibits M2 polarization of co‐cultured macrophages. (A) Schematic illustration of the co‐culturing model for macrophages and LUAD cells transfected with Cy3‐labeled miR‐19b‐3p. (B) Fluorescence microscopy images of Cy3‐miR‐19b‐3p fluorescence in THP1 (Mφ) cells cultured without (Control) or with A549 LUAD cells transfected with Cy3‐miR‐19b‐3p. FITC‐Phalloidin (green) and Cy3 (red) were adopted for labeling cytoskeleton and miR‐19b‐3p, respectively. Scale bar: 10μm. (C) THP1 (Mφ) cells were co‐cultured with untreated LUAD cells (A549 or H1975) or LUAD cells transfected with NC inhibitors (A549/NC inhibitors or H1975/NC inhibitors) or miR‐19b‐3p inhibitors (A549/miR‐19b‐3p inhibitors or H1975/miR‐19b‐3p inhibitors), or without co‐culturing (NC). qRT‐PCR was adopted to detect the levels of M2 markers in THP1 (Mφ) cells of each group of co‐culturing system. (D) Flow cytometry was applied to measure CD206 +CD11b + macrophages in each group of co‐culturing system. THP1 (Mφ), macrophages induced from THP1 cells. Data are presented as mean ± SD of at least three independent experiments. **p < 0.01

To identify exosomal miR‐19b‐3p function in M2 polarization, we cocultured THP1 (Mφ) cells with LUAD cells transfected with Cy3‐miR‐19b‐3p (Figure 2A). After 24 h of the coculture, we monitored the fluorescence of Cy3‐miR‐19b‐3p in THP1 (Mφ) cells in the coculture group instead of monoculture group, indicating that miR‐19b‐3p was transported from LUAD cells to THP1 (Mφ) cells (Figure 2B). MiR‐19b‐3p in LUAD cells and exosomes derived from LUAD cells was declined by miR‐19b‐3p inhibitor (Supporting information Figure S7C). The mRNA levels of M2 markers were examined in THP1 (Mφ) cells cocultured with LUAD cells transfected with or without miR‐19b‐3p inhibitors. Notably, we observed that M2 markers were increased in THP1 (Mφ) cells of A549/H1975 co‐culture group and such increase was reversed in THP1 (Mφ) cells of A549/miR‐19b‐3p inhibitors group or H1975/miR‐19b‐3p inhibitors group (Figure 2C). Additionally, levels of M1 markers, including TNF‐α, NOS2, CXCL9, and CXCL10, were not changed in THP1 (Mφ) cells of groups mentioned above (Supporting information Figure S9B). Consistently, the CD11b+CD206+ ratio was enhanced in THP1 (Mφ) cells cocultured with LUAD cells but such enhancement was reversed when the THP1 (Mφ) cells were cocultured with miR‐19b‐3p‐silenced LUAD cells (Figure 2D). Thus, the knockdown of miR‐19b‐3p in LUAD cells inhibits M2 polarization in THP1 (Mφ) cells.

### 3.4 Exosomal miR‐19b‐3p promotes M2 polarization through PTPRD/STAT3 axis

**FIGURE 3:**
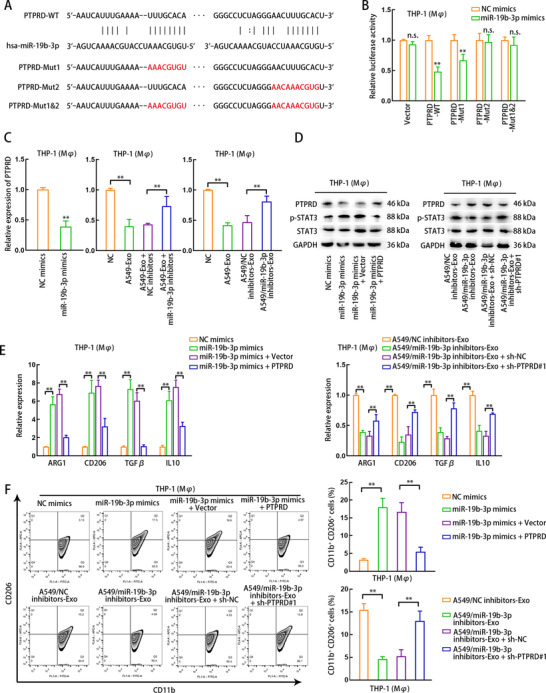
Exosomal miR‐19b‐3p facilitates M2 macrophage polarization through regulating PTPRD/STAT3. (A) Putative binding sites between PTPRD mRNA and miR‐19b‐3p. The nucleotides highlighted in red stand for mutant binding sites were designed for luciferase reporter assay. (B) Luciferase reporter assay demonstrated the change of relative luciferase activity of each construct harboring wild‐type or mutant PTPRD sequence or empty vector in response to miR‐19b‐3p mimic transfection in THP1 (Mφ) cells. (C) qRT‐PCR data of PTPRD level in THP1 (Mφ) cells. THP1 (Mφ) cells were transfected with NC mimic or miR‐19b‐3p mimic; treated without (NC) or with A549‐derived exosomes (A549‐Exo), treated with A549‐Exo and transfected with NC inhibitors (A549‐Exo+NC inhibitors) or miR‐19b‐3p inhibitors (A549‐Exo+miR‐19b‐3p inhibitors); or treated with exosomes from A549 cells transfected with NC inhibitor (A549/NC inhibitors‐Exo) or miR‐19b‐3p (A549/miR‐19b‐3p inhibitors‐Exo). (D) Western blot presented PTPRD expression in THP1 (Mφ) cells transfected with NC mimics, miR‐19b‐3p mimics, miR‐19b‐3p mimics+Vector, or miR‐19b‐3p+PTPRD; in THP1 (Mφ) cells treated with exosomes derived from A549 cells transfected with NC inhibitors (A549/NC inhibitors‐Exo) or miR‐19b‐3p inhibitors (A549/miR‐19b‐3p inhibitors‐Exo); or in THP1 (Mφ) cells treated with A549/miR‐19b‐3p inhibitors‐Exo and transfected with sh‐NC (A549/miR‐19b‐3p inhibitors‐Exo+sh‐NC), or treated with A549/miR‐19b‐3p inhibitors‐Exo and transfected with sh‐PTPRD#1 (A549/miR‐19b‐3p inhibitors‐Exo+sh‐PTPRD#1). (E) qRT‐PCR demonstrated the level of M2 markers in THP1 (Mφ) cells of each group mentioned above. (F) Flow cytometry was applied to measure CD206 +CD11b + macrophages in each group. THP1 (Mφ), macrophages induced from THP1 cells. A549‐Exo, exosomes derived from A549 cells. Data are presented as mean ± SD of at least three independent experiments. **p < 0.01, n.s., no significance

Later, we probed whether miR‐19b‐3p regulated PTPRD/STAT3 axis in THP1 (Mφ) cells to affect M2 polarization. The miR‐19b‐3p binding sites (wild‐ or mutant‐type) in PTPRD were predicted and illustrated in Figure 3A. The influence of miR‐19b‐3p on PTPRD at the potential sites was further proven by luciferase reporter assay. Additionally, we validated miR‐19b‐3p overexpression in THP1 (Mφ) cells, LUAD cells and LUAD cell‐derived exosomes (Supporting information Figure S7D). According to the data shown in Figure 3B, miR‐19b‐3p mimics abrogated the luciferase activity of PTPRD‐WT and PTPRD‐Mut1 reporters (with only site1 mutated) but that of PTPRD‐Mut2 (with only site2 mutated) and PTPRD‐Mut1+2 (with both site1 and site2 mutated) reporters showed no changes in luciferase activity under miR‐19b‐3p overexpression, implying that the function of miR‐19b‐3p on PTPRD can be abrogated only when site2 was mutated, which meant that miR‐19b‐3p affected PTPRD by binding at site2. Unsurprisingly, the mRNA level of PTPRD was negatively affected by miR‐19b‐3p mimics in THP1 (Mφ) cells (Figure 3C, left). Additionally, transfecting miR‐19b‐3p inhibitors into THP1 (Mφ) cells or LUAD cells both abrogated the effect of LUAD cell‐derived exosomes on inhibiting PTPRD level in THP1 (Mφ) cells (Figure 3C, middle and right). Next, the decrease of PTPRD protein level and the increase of p‐STAT3 level were obtained in THP1 (Mφ) cells transfected with miR‐19b‐3p mimics, but these results were partially rescued by PTPRD overexpression (Figure 3D, left). We also observed that PTPRD protein was increased but p‐STAT3 was decreased in THP1 (Mφ) cells treated with exosomes derived from A549 cells transduced with miR‐19b‐3p inhibitors, but these effects were partly countervailed by the silencing PTPRD in THP1 (Mφ) cells (Figure 3D, right). The levels of M2 polarization markers were respectively examined in transfected THP1 (Mφ) cells or cocultured THP1 (Mφ) cells. The mRNA levels of four M2 markers were upregulated in THP1 (Mφ) cells with miR‐19b‐3p overexpression, but were downregulated after the cooverexpression of PTPRD (Figure 3E, left). However, the levels of four M2 markers were reduced overtly in THP1 (Mφ) cells treated with exosomes secreted by miR‐19b‐3p‐silenced A549 cells, but such effect was partially abolished by PTPRD silencing in THP1 (Mφ) cells (Figure 3E, right). Similar results were achieved when monitoring CD206 +CD11b+ ratio of THP1 (Mφ) cells by flow cytometry analysis (Figure 3F). Collectively, LUAD cell‐derived exosomal miR‐19b‐3p promotes M2 polarization by targeting PTPRD and enhancing STAT3 phosphorylation.

### 3.5 Suppression of M2 polarization induced by miR‐19b‐3p silencing inhibits LUAD tumor metastasis

**FIGURE 4:**
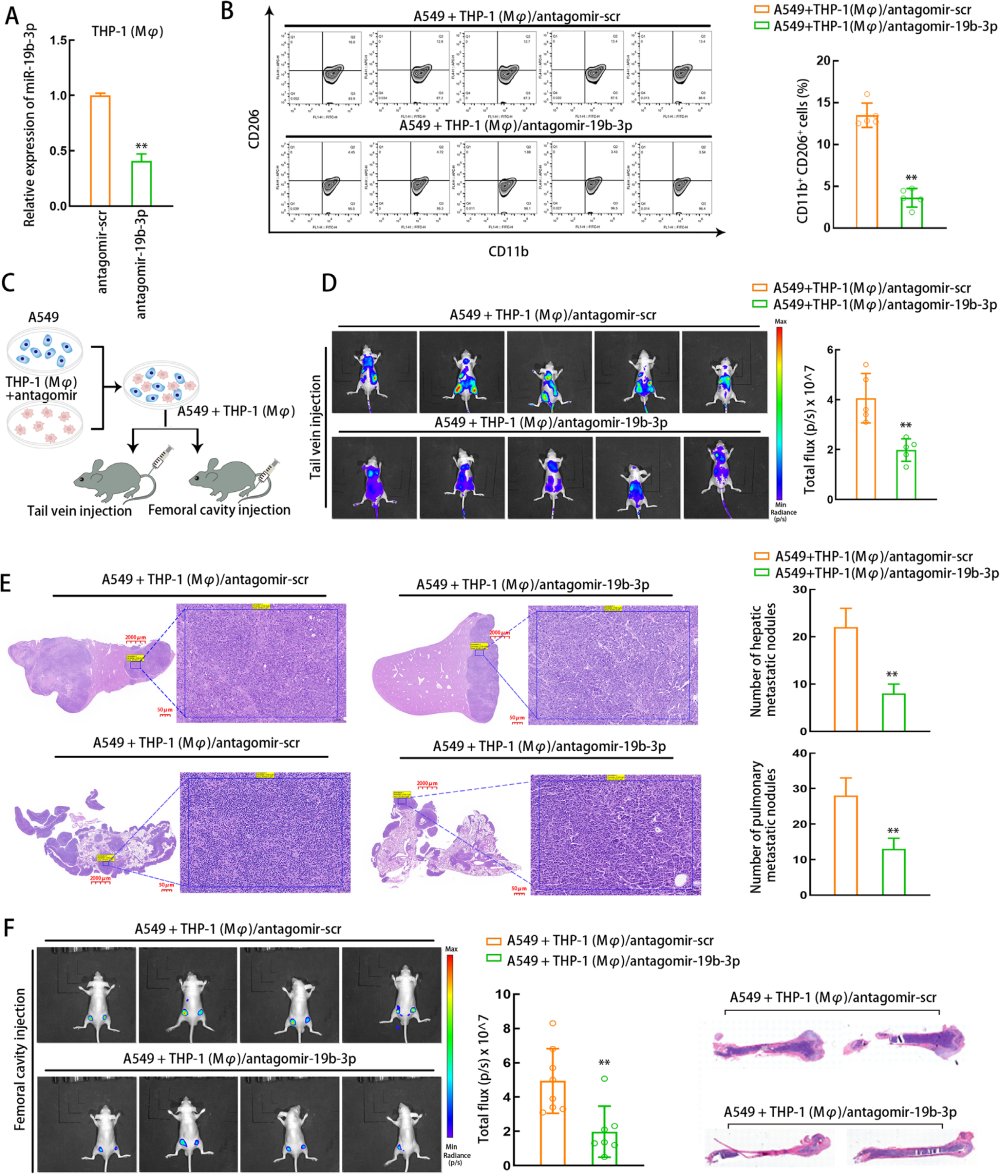
miR‐19b‐3p knockdown suppresses M2 macrophage polarization and inhibits LUAD tumor metastasis in vivo. (A) The antagomir‐mediated knockdown efficiency of miR‐19b‐3p was detected by qRT‐PCR in THP1 (Mφ) cells. (B) Transfected THP1 (Mφ) cells were co‐cultured with A549 cells and flow cytometry results presented the ratio of CD206 +CD11b + THP1 (Mφ) cells after co‐culture. (C) Schematic illustration presented that transfected THP1 (Mφ) cells with different transfections were mixed with A549 LUAD cells and injected into mice from tail vein or femoral cavity. (D‐E) In vivo bioluminescent imaging assay (D) and H&E staining (E) for hepatic and pulmonary metastases for mice in each group of tail vein injection (n = 5 of each group). Scale bar: 2000 μm, 50 μm (enlarged). (F) In vivo bioluminescent imaging assay and H&E staining for osseous metastasis in mice of each group with femoral cavity injection (n = 4 of each group). THP1 (Mφ), macrophages induced from THP1 cells. Data are presented as mean ± SD of at least three independent experiments. **p < 0.01

Subsequently, we verified the downregulation of miR‐19b‐3p in THP1 (Mφ) cells after THP1 (Mφ) cells were transfected with antagomir‐19b‐3p and cocultured with A549 cells (Figure 4A). Flow cytometry results displayed that antagomir‐19b‐3p reduced the ratio of CD11b+CD206 + THP1 (Mφ) cells cocultured with A549 cells (Figure 4B). Transfected THP1 (Mφ) cells and A549 cells were mixed and injected into mice from tail vein or femoral cavity (Figure 4C). After the tail vein injection, we observed that hepatic metastasis and pulmonary metastasis were attenuated in mice of A549 + THP1 (Mφ)/antagomir‐19b‐3p group versus A549 +THP1 (Mφ)/antagomir/src group (Figures 4D–4E). Furthermore, osseous metastatic model was constructed through femoral cavity injection. We observed that the osseous metastasis was also attenuated in mice of A549 + THP1 (Mφ)/antagomir‐19b‐3p group versus A549 + THP1 (Mφ)/antagomir/src group (Figure 4F). These data indicated that silencing of miR‐19b‐3p reverses M2 polarization and, thus, suppresses LUAD tumor metastasis in vivo.

### 3.6 Exosomes secreted by M2‐polarized macrophages facilitates LUAD cell migration and invasion

M2 polarization is an important indicator in tumor progression. Here, we analyzed whether M2‐polarized macrophages were involved in LUAD cell migration and invasion. A549 cells were cultured in the CM from THP1 (Mφ) cells respectively treated with NC mimics, miR‐19b‐3p mimics, miR‐19b‐3p mimics + DMSO, or miR‐19b‐3p mimics + GW4869. Experimental results revealed that the migratory and invasive abilities of A549 cells were strengthened by the coculture with THP1 (Mφ) cells containing miR‐19b‐3p mimics, but was weakened again by the treatment with GW4869 (Supporting information Figure S3A‐B), indicating that M2‐polarized macrophages promoted LUAD cell migration and invasion through secreting exosomes. Additionally, the viability of A549 cells was enhanced when cultured with CM from miR‐19b‐3p‐upreglated THP1 (Mφ) cells, and this effect was abrogated when the CM was added with GW4869 (Figure S3C).

Then, we observed the exosomes extracted from THP1 (Mφ) cells with or without miR‐19b‐3p overexpression using TEM (Supporting information Figure S3D). The fluorescence of DiO in A549 cells treated with THP1 (Mφ)‐derived exosomes with NC mimics or miR‐19b‐3p mimics was monitored by fluorescence microscopy. Consequently, the DiO florescence was shown in A549 cells treated with the THP1 (Mφ) exosomes, and the increase of DiO+ A549 cells in 24 h was facilitated by the treatment with THP1 (Mφ) exosomes with miR‐19b‐3p overexpression compared with treatment with THP1 (Mφ) exosomes with NC mimic, indicating that A549 cells absorbed the exosomes secreted by THP1 (Mφ) cells (Supporting information Figure S3E). Next, we collected exosomes from THP1 (Mφ) cells transduced with NC mimics or miR‐19b‐3p mimics, respectively. The exosomes of two groups were used to treat A549 cells separately. In consequence, we determined A549 cell migration and invasion were strengthened in THP1 (Mφ)/miR‐19b‐3p mimics‐Exo group compared with THP1 (Mφ)/NC mimics‐Exo group (Supporting information Figure S3F‐G). These findings revealed that exosomes secreted by M2‐polarized macrophages facilitates LUAD cell migration and invasion in a miR‐19b‐3p‐dependent manner. However, what molecules packaged in exosomes actually influenced LUAD cell migration and invasion was unknown.

### 3.7 MiR‐19b‐3p‐induced M2 macrophages secrets exosomes to induce YAP dephosphorylation by LATS2 ubiquitination in LUAD cells

**FIGURE 5:**
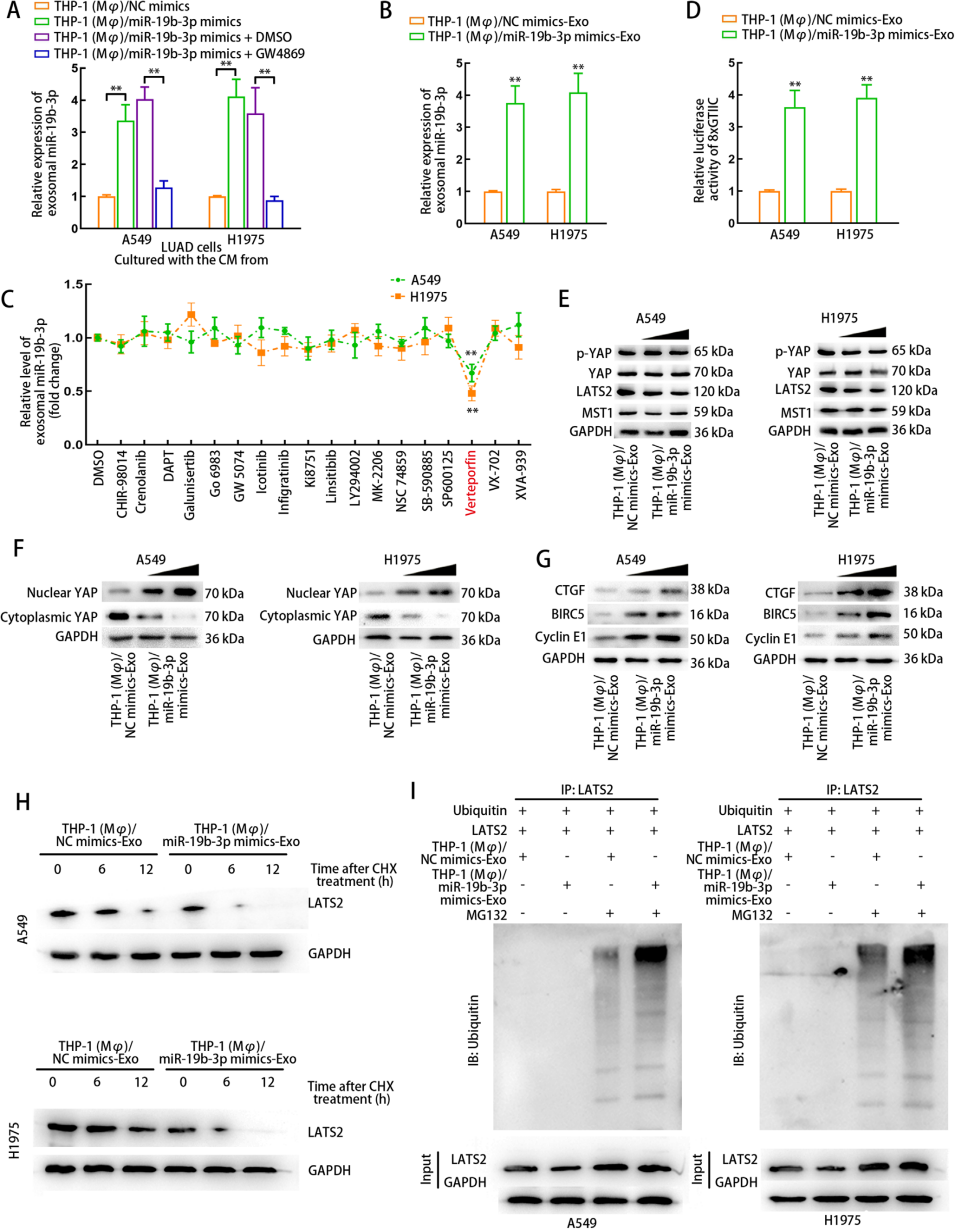
Exosomes from miR‐19b‐3p‐induced macrophages regulates Hippo signaling pathway by inducing LATS2 ubiquitination in LUAD cells. (A) A549 and H1975 cells were cultured with the CM from THP1 (Mφ) treated with NC mimics, miR‐19b‐3p mimics, miR‐19b‐3p mimics + DMSO, or miR‐19b‐3p mimics + GW4869. miR‐19b‐3p level in LUAD cell‐derived exosomes of each group was detected using qRT‐PCR. (B) A549 and H1975 cells were treated with THP1 (Mφ)/NC mimics‐Exo or THP1 (Mφ)/miR‐19b‐3p mimics‐Exo. miR‐19b‐3p level in LUAD cell‐derived exosomes of each group was detected using qRT‐PCR. (C) A series of qRT‐PCR analyses illustrated the fold changes of exosomal miR‐19b‐3p levels from A549 and H1975 cells treated with various small‐molecule inhibitors. DMSO treatment was regarded as a control. (D) Luciferase reporter assay detected YAP signaling in A549 and H1975 cells treated with THP1 (Mφ)/NC mimics‐Exo or THP1 (Mφ)/miR‐19b‐3p mimics‐Exo using 8xGTIIC‐luciferase YAP/TAZ reporters. (E) The effects of rising dose of THP1 (Mφ)/miR‐19b‐3p mimics‐Exo on the levels of p‐YAP, YAP, LATS2 and MST1 in LUAD cells were evaluated using western blot. (F) The effects of rising dose of THP1 (Mφ)/miR‐19b‐3p mimics‐Exo on the levels of nuclear or cytoplasmic YAP in LUAD cells were evaluated using western blot. (G) The effects of rising dose of THP1 (Mφ)/miR‐19b‐3p mimics‐Exo on the levels of YAP downstream genes in LUAD cells were evaluated using western blot. (H) CHX chase assay evaluated the stability of LATS2 protein in LUAD cells cultured with the exosomes of each group. Western blot was used to detect LATS2 expression in LUAD cells of each group after CHX treatment at different time points. (I) Ubiquitination assay evaluated the ubiquitination level of LATS2 protein in LUAD cells cultured with the exosomes of each group. THP1 (Mφ), macrophages induced from THP1 cells. THP1 (Mφ)/NC mimics‐Exo or THP1 (Mφ)/miR‐19b‐3p mimics‐Exo, exosomes derived from THP1 (Mφ) transfected with NC mimics or miR‐19b‐3p mimics. Data are presented as mean ± SD of at least three independent experiments. **p < 0.01

Then, we tried to explain through what mechanism miR‐19b‐3p‐induced M2 macrophages affected LUAD cells. First, we determined the exosomal miR‐19b‐3p expression from LUAD cells in the CM from THP1 (Mφ) cells separately treated with NC mimics, miR‐19b‐3p mimics, miR‐19b‐3p mimics + DMSO, or miR‐19b‐3p mimics + GW4869. Intriguingly, miR‐19b‐3p was upregulated in LUAD cell‐derived exosomes when LUAD cells were cultured with CM of THP1 (Mφ) cells with miR‐19b‐3p‐overexpression, but the expression of LUAD cell‐derived exosomal miR‐19b‐3p was downregulated again after LUAD cells were treated with CM from THP1 (Mφ) cells cotreated with miR‐19b‐3p mimics and GW4869 (Figure 5A). In addition, we confirmed that when overexpressing miR‐19b‐3p in THP1 (Mφ) cells, the exosomes from THP1 (Mφ) cells did not present miR‐19b‐3p upregulation (Supporting information Figure S8C). This result excluded the possibility that the upregulation of exosomal miR‐19b‐3p level in coculture system came from the exosomes of THP1 (Mφ) cells. Interestingly, the miR‐19b‐3p expression of LUAD cells was not altered when LUAD cells were cultured with the CM from miR‐19b‐3p‐induced THP1 (Mφ) cells treated without or with GW4869 (Supporting information Figure S7E). Additionally, miR‐19b‐3p expression was higher in exosomes derived from LUAD cells after LUAD cells were treated with exosomes derived from THP1 (Mφ) cells transfected with miR‐19b‐3p mimics or NC mimics (THP1 (Mφ)/miR‐19b‐3p mimics‐Exo or THP1 (Mφ)/NC mimics‐Exo) (Figure 5B). Therefore, these data indicated that the THP1 (Mφ) cells treated with miR‐19b‐3p could affect exosomal miR‐19b‐3p level derived from LUAD cells in return, but could not affect miR‐19b‐3p expression of LUAD cells.

To identify certain clinical signaling pathway involved in the regulation on LUAD cell‐derived exosomal miR‐19b‐3p level, we added the inhibitors specific to various signaling pathways into LUAD cells. As a result, only Verteporfin (YAP inhibitor) could downregulate miR‐19b‐3p level in LUAD cell‐derived exosomes (Figure 5C), indicating that VP regulated exosomal miR‐19b‐3p level from LUAD cells. Thus, we wondered whether exosomes derived from THP1 (Mφ) cells with miR‐19b‐3p overexpression can affect Hippo/YAP signaling to regulate the level of LUAD cell‐derived exosomal miR‐19b‐3p. To prove this hypothesis, we transfected 8x GTIIC‐luciferase reporter into LUAD cells to detect YAP signaling activity in LUAD cells. Consequently, the enhanced luciferase activity of 8x GTIIC‐luciferase reporter indicated that YAP signaling in LUAD cells was activated by the treatment of THP1 (Mφ) cell‐derived exosomes with miR‐19b‐3p overexpression (Figure 5D). Western blot analysis revealed that p‐YAP and LATS2 levels were gradually decreased in LUAD cells treated with a gradually increasing dose of exosomes secreted by THP1 (Mφ) cells with miR‐19b‐3p overexpression (Figure 5E). Importantly, accumulation of nuclear YAP and levels of targets downstream of YAP were increased in LUAD cells by the treatment of exosomes secreted from miR‐19b‐3p‐overexpressed THP1 (Mφ) cells (Figure 5F and G). Therefore, we concluded that M2‐polarized THP1 (Mφ) cells secreted exosomes to promote exosomal miR‐19b‐3p level from LUAD cells through activating YAP signaling.

Furthermore, we detected whether THP1 (Mφ) cell‐derived exosomes of the indicated groups regulated YAP nuclear translocation in LUAD cells by modulating LATS2 protein. After the treatment of THP1 (Mφ) cell‐derived exosomes of different groups, LUAD cells were treated with CHX. Then, LATS2 protein level was detected at different time points. Consequently, the half‐life of LATS2 protein was shortened in LUAD cells incubated with exosomes secreted by miR‐19b‐3p‐upregulated THP1 (Mφ) cells (Figure 5H). Therefore, we proceeded to explore the effect of miR‐19b‐3p overexpressed THP1 (Mφ) cell‐derived exosomes on LATS2 ubiquitination in LUAD cells. LATS2 ubiquitination was enhanced in LUAD cells treated with exosomes secreted by miR‐19b‐3p‐upregulated THP1 (Mφ) cells (Figure 5I). Therefore, miR‐19b‐3p‐induced M2 macrophages secretes exosomes to induce YAP dephosphorylation by LATS2 ubiquitination in LUAD cells.

### 3.8 M2‐polarized macrophages transmits exosomal LINC00273 to induce LATS2 degradation in LUAD cells by recruiting NEDD4

**FIGURE 6:**
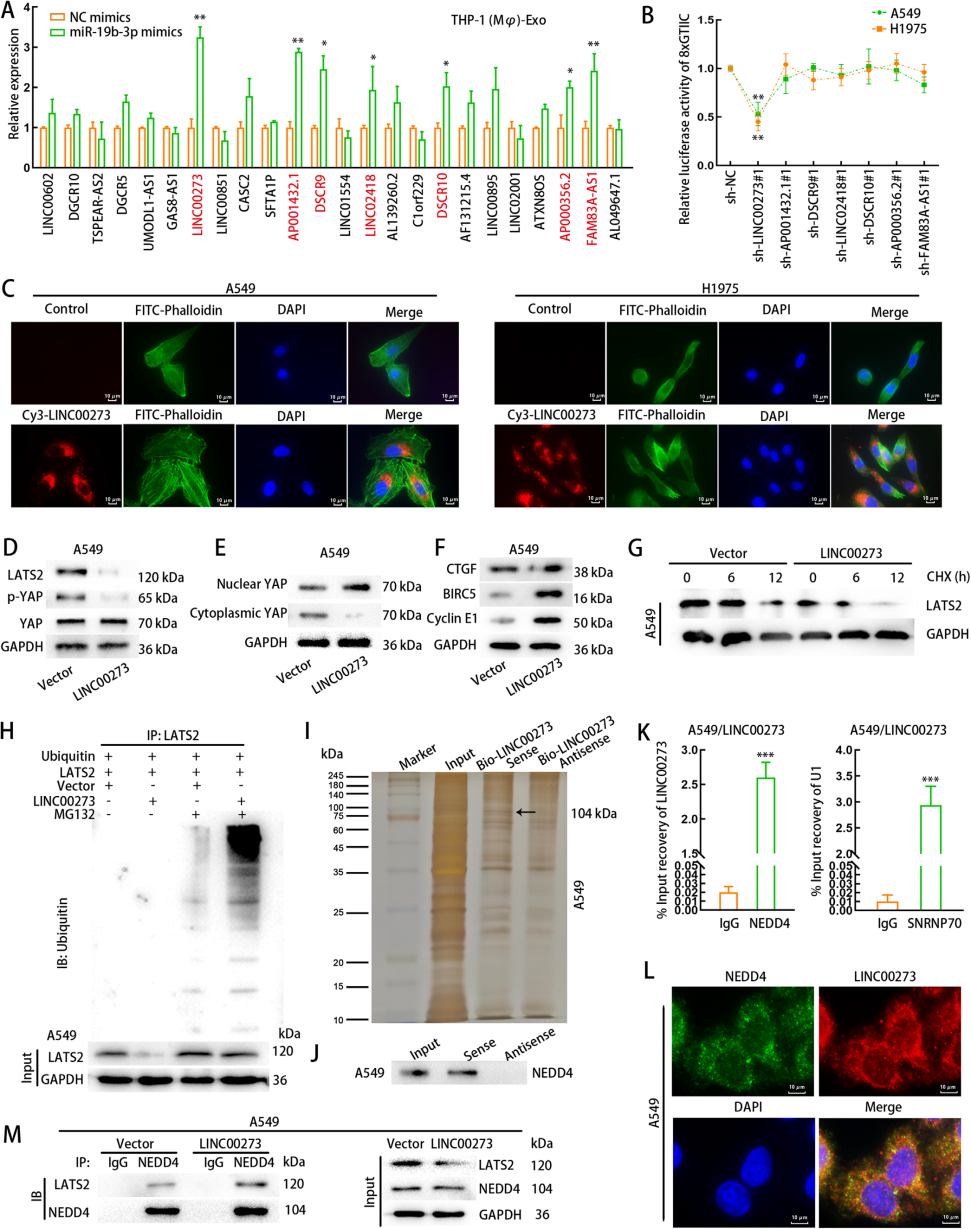
Macrophage‐transferred exosomal LINC00273 regulates Hippo signaling pathway by recruiting NEDD4 in LUAD cells. (A) Twenty‐four highly expressed lncRNAs (log_2_FC > 1) in lung cancer were searched out from BBCancer database. After the transfection of NC mimics or miR‐19b‐3p mimics, exosomal lncRNA levels from THP1 (Mφ) were detected, and 7 significantly up‐regulated lncRNAs by miR‐19b‐3p mimics were selected. (B) A series of luciferase reporter assay illustrated the fold change of YAP signaling in A549 and H1975 cells were treated with THP1 (Mφ)‐derived exosomes and were subjected to the knockdown of each lncRNA selected above or sh‐NC. (C) THP1 (Mφ) cells were transfected with Cy3‐LINC00273 (red) and the exosomes derived from the transfected THP1 (Mφ) cells were added to the medium of LUAD cells for incubation. Fluorescence microscopy images illustrated the transportation of THP1 (Mφ)‐derived exosomal LINC00273 to LUAD cells. FITC Phalloidin (green) and Cy3 (red) were adopted for labeling cytoskeleton of LUAD cells and LINC00273, respectively. Scale bar: 10μm. (D) LATS2, p‐YAP and YAP levels in A549 cells transfected with pcDNA3.1 or pcDNA3.1/LINC00273 were evaluated using western blot. (E) Nuclear and cytoplasmic YAP levels in A549 cells transfected with pcDNA3.1 or pcDNA3.1/LINC00273 were evaluated using western blot. (F) The levels of YAP downstream genes in A549 cells transfected with pcDNA3.1 or pcDNA3.1/LINC00273 were evaluated using western blot. (G) CHX chase assay evaluated the stability of LATS2 protein in A549 cells transfected with pcDNA3.1 or pcDNA3.1/LINC00273. (H) Ubiquitination assay evaluated the ubiquitination level of LATS2 protein in A549 cells transfected with pcDNA3.1 or pcDNA3.1/LINC00273. (I) Proteins recruited by LINC00273 in A549 cells were assessed by mass spectrometric analyses following RNA pull‐down assay. The protein band corresponding to NEDD4 was indicated in the image of SDS‐PAGE gel. (J) The pull‐down enrichment product of Bio‐LINC00273‐Sense or Antisense probe was subjected to western blot to determine the existence of NEDD4. (K) The binding capacity between NEDD4 and LINC00273 was confirmed by RIP assay in A549 cells with LINC00273 overexpression. U1 RNA co‐immunoprecipitated with SNRNP70 was adopted as the positive control. (L) The combination of immunofluorescence and FISH illustrated the overlapped distribution of NEDD4 (green) and LINC00273 (red) in cytoplasm of A549 cells with LINC00273 overexpression. Scale bar: 10μm. (M) Co‐IP assay detected the level of LATS2 or NEDD4 co‐immunoprecipitated with anti‐NEDD4 (anti‐IgG as negative control) in A549 cells transfected with pcDNA3.1 or pcDNA3.1/LINC00273. THP1 (Mφ), macrophages induced from THP1 cells. Data are presented as mean ± SD of at least three independent experiments. *p < 0.05, **p < 0.01, ***p < 0.001

As previously reported, exosomes secreted by M2‐polarized macrophages can transmit lncRNA into recipient cells.^60^ Here, we suspected that M2/exosomes probably exerted oncogenic functions in LUAD tumor microenvironment depending on the secretion of a certain lncRNA. At first, the levels of 24 lncRNA upregulated (FC > 2) in the blood samples of LUAD patients in BBCancer database were examined in exosomes secreted by THP1 (Mφ) cells with or without miR‐19b‐3p overexpression. Seven of these lncRNAs were upregulated in THP1 (Mφ)‐derived exosomes with miR‐19b‐3p overexpression (Figure 6A). Furthermore, we separately silenced these seven lncRNAs in LUAD cells to test the luciferase activity of 8x GTIIC reporter vector. The luciferase activity decreased in response to LINC00273 silencing in LUAD cells treated with THP1 (Mφ)‐derived exosomes with miR‐19b‐3p overexpression (Figure 6B), indicating LINC00273 regulated YAP pathway in LUAD cells.

Later, miR‐19b‐3p overexpression in THP1 (Mφ) cells led to the upregulation of LINC00273 in THP1 (Mφ) cells (Supporting information Figure S8A). Also, LINC00273 level was upregulated in THP1 (Mφ) cell‐derived exosomes when THP1 (Mφ) cells were transduced with miR‐19b‐3p mimics (Supporting information Figure S8B). Directly overexpressing miR‐19b‐3p in LUAD cells failed to alter LINC00273 level in LUAD cells (Supporting information Figure S8D). These data indicated that miR‐19b‐3p only functioned as LUAD cell‐derived exosomal miRNA. Also, we found miR‐19b‐3p‐overexpressed THP1 (Mφ) cells presented higher LINC00273 level than LUAD cells (Supporting information Figure S8E), indicating the possibility that M2‐polarized macrophages transferred exosomal LINC00273 to LUAD cells. Unsurprisingly, the Cy3‐LINC00273 fluorescence was detected in LUAD cells after treatment with THP1 (Mφ)‐derived exosomes transfected with Cy3‐LINC00273 for 24 hours, whereas A549 cells without exosome treatment presented no fluorescence (Figure 6C), confirming that LINC00273 was transported from THP1 (Mφ)‐derived exosomes to LUAD cells.

Subsequently, the levels of p‐YAP and LATS2 were decreased in LUAD cells after overexpressing LINC00273 (Figure 6D). Meanwhile, LINC00273 overexpression promoted the nuclear accumulation of YAP protein and upregulated the protein levels of YAP downstream targets (Figures 6E–6F). Moreover, upregulation of LINC00273 shortened the half‐life of LATS2 protein under CHX treatment (Figure 6G). LATS2 ubiquitination was also increased by LINC00273 overexpression in LUAD cells (Figure 6H). Subsequently, RNA pull‐down with mass spectrometry were conducted to uncover whether LINC00273 modulated LATS2 ubiquitination through interacting with ubiquitination‐related proteins.

Then, we explored how LINC00273 regulated LATS2 ubiquitination. As shown in Figure 6I and Figure S7F, we determined by pulldown and mass spectrometry that LINC00273 could interact with NEDD4 (an E3 ubiquitin ligase). Western blot analysis of pull‐down products revealed that NEDD4 was efficiently pulled down by bio‐LINC00273 sense (Figure 6J). The interaction between LINC00273 and NEDD4 was further proven by RIP assay (Figure 6K). Through FISH assay and immunofluorescence, we identified that LINC00273 interacted with NEDD4 at the cytoplasm of A549 cells (Figure 6L). According to the data of Co‐IP assay, NEDD4 could interact with LATS2 in LUAD cells, and this interaction was increased by LINC00273 overexpression, but the input NEDD4 level was not changed (Figure 6M). Together, M2‐polarized macrophages transmits exosomal LINC00273 to induce LATS2 degradation in LUAD cells through recruiting NEDD4.

### 3.9 Knockdown of LINC00273 suppresses LUAD tumor metastasis

**FIGURE 7:**
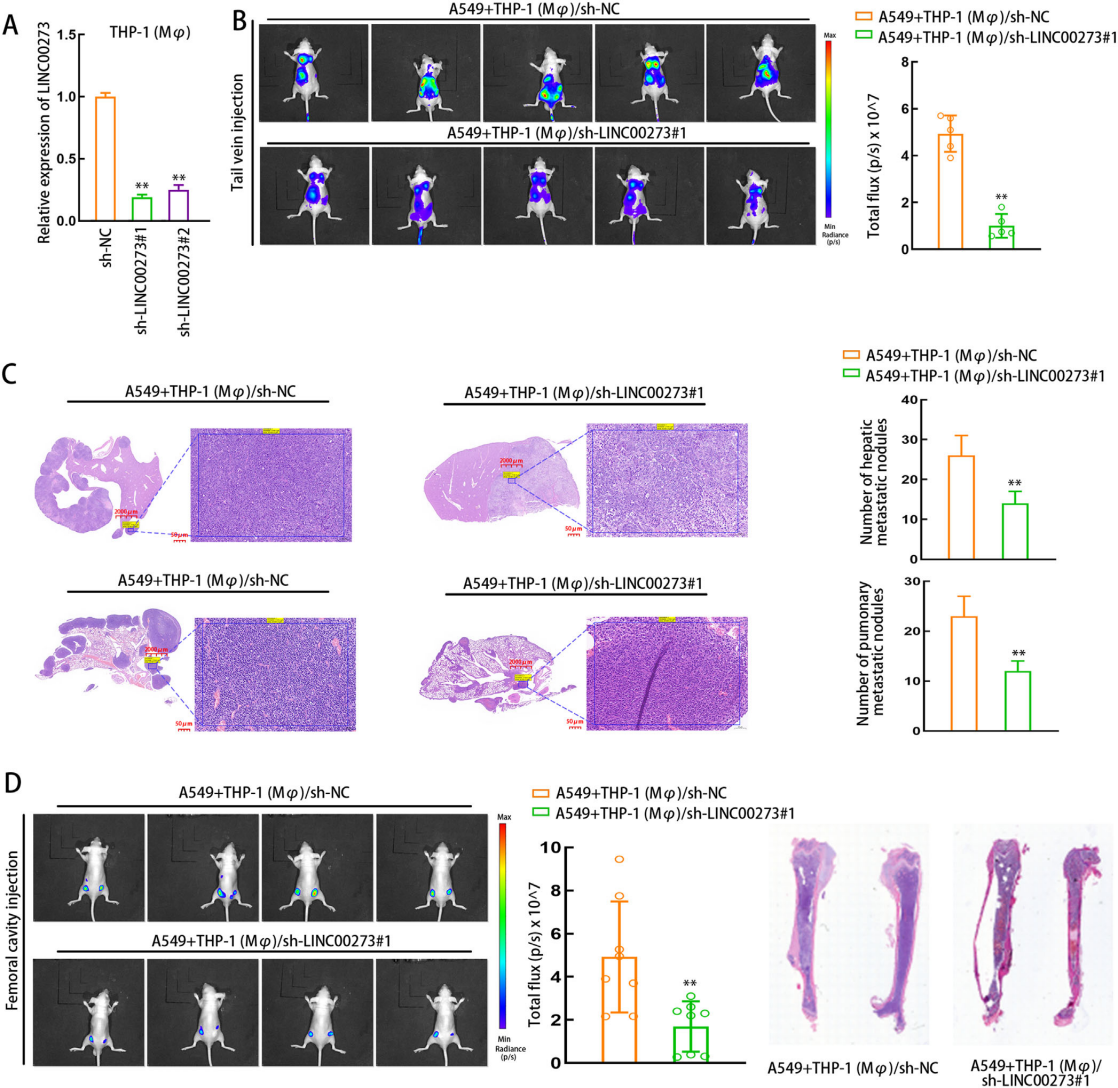
LINC00273 knockdown inhibits LUAD tumor metastasis in vivo. (A) The knockdown efficiency of LINC00273 was detected by qRT‐PCR in THP1 (Mφ). (B‐C) In vivo bioluminescent imaging assay (B) and H&E staining (C) for hepatic and pulmonary metastases for mice in each group of tail vein injection (n = 5 of each group). Scale bar: 2000 μm, 50 μm (enlarged). (D) In vivo bioluminescent imaging assay and H&E staining for osseous metastasis in mice of each group with femoral cavity injection (n = 4 of each group). Scale bar: 1000 μm, 50 μm (enlarged). THP1 (Mφ), macrophages induced from THP1 cells. Data are presented as mean ± SD of at least three independent experiments. **p < 0.01

To check the potential function of M2 macrophage‐derived exosomal LINC00273 on LUAD metastasis, we silenced LINC00273 in THP1 (Mφ) cells. Results confirmed that LINC00273 level declined in THP1 (Mφ) cells transfected with sh‐LINC00273#1/2 (Figure 7A). Then, the two types of cells were mixed and injected into mice from tail vein or femoral cavity. According to in vivo metastasis assay, LINC00273 silencing led to the depletion of hepatic metastasis and pulmonary metastasis (Figure 7B and C). By observing the metastasis in mice subjected with femoral cavity injection, we confirmed the inhibitory effect of LINC00273 knockdown on the osseous metastasis (Figure 7D). These data suggested that LINC00273 inhibits LUAD tumor metastasis.

### 3.10 STAT3 induces transcriptional activation of LINC00273 in macrophages

The abovementioned data have shown that miR‐19b‐3p promoted STAT3 phosphorylation to induce M2 polarization of macrophages. STAT3 commonly worked as a transcription activator in cells, thus, we hypothesized that LINC00273 might be transcriptionally regulated by STAT3 in M2 macrophages. The expression of LINC00273 was found to be enhanced in THP1 (Mφ) cells by A549‐exosome treatment or miR‐19b‐3p mimics, but the results were reversed in THP1 (Mφ) cells treated with NSC 74859 (also named S3I‐201, which can prevent STAT3 from binding to DNA sequence) (Supporting information Figure S4A‐B). Next, we found that STAT3 overexpression led to LINC00273 and STAT3 upregulation in THP1 (Mφ) cells (Supporting information Figure S4C and D). Thereafter, we identified that STAT3 bound to LINC00273 promoter at ‐2000 ∼ ‐1000 region (Supporting Information Figure S4E). The DNA motif and binding sequences were predicted by using JASPAR tool based to analyze LINC00273 promoter sequences obtained from UCSC (Supporting Information Figure S4F). Further, luciferase activity analysis indicated that both two binding sequences were responsible for the transcriptional activation of LINC00273 (Supporting information Figure S4G‐H). Finally, we observed from ChIP assay that the abundance of LINC00273 promoter in STAT3 precipitates was increased by overexpressing miR‐19b‐3p but was decreased by NSC 74959 (Supporting information Figure S4I).

### 3.11 LINC00273 increased the binding of RBMX to miR‐19b‐3p and promotes the RBMX‐mediated packaging of miR‐19b‐3p into LUAD cell‐derived exosomes

**FIGURE 8:**
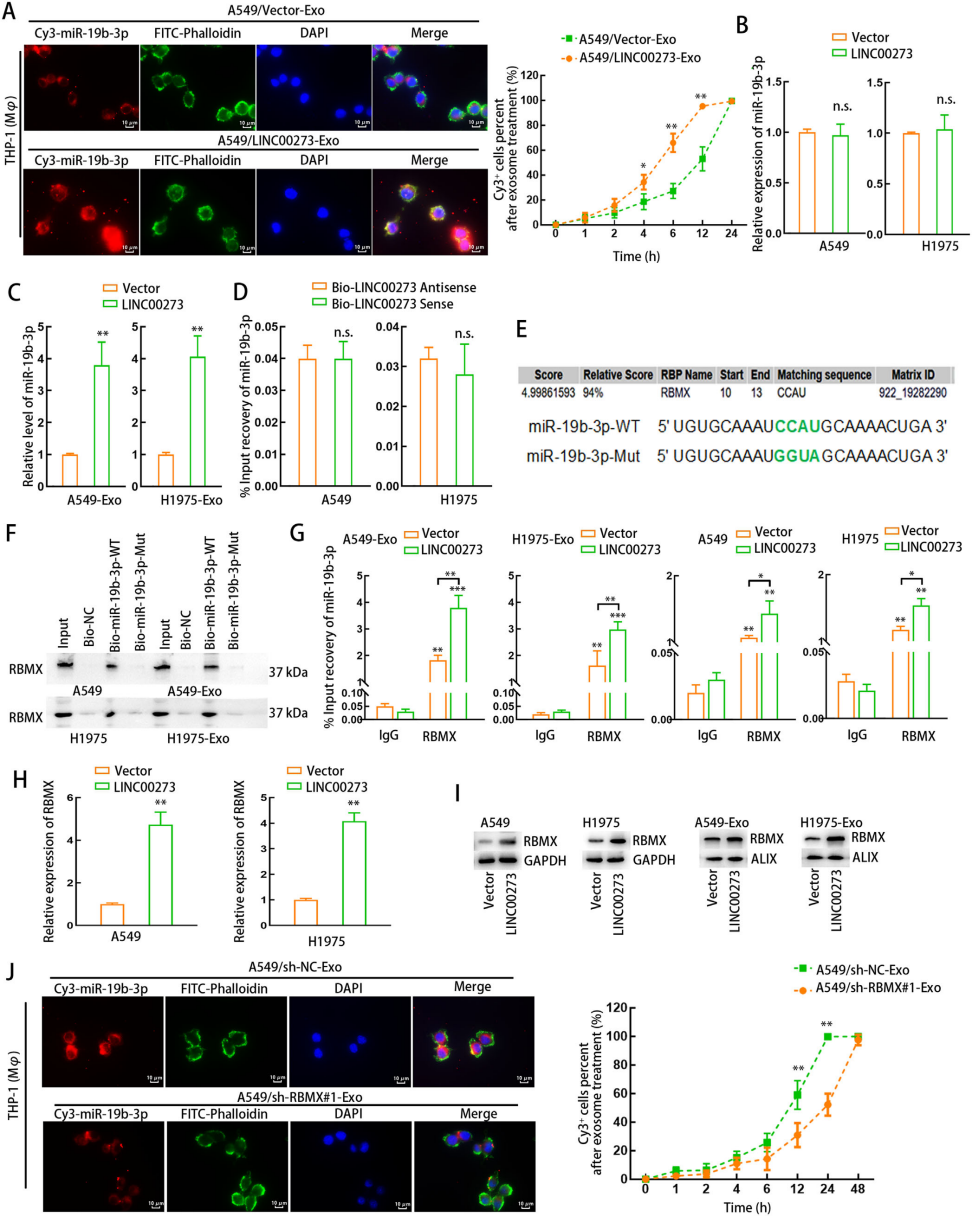
LINC00273 facilitates exosomal miR‐19b‐3p secretion via up‐regulating RBMX. (A) Exosomes were extracted from A549 cells transfected with Cy3‐miR‐19b‐3p and pcDNA3.1 or pcDNA3.1/LINC00273. Fluorescence microscopy images detected the Cy3‐miR‐19b‐3p fluorescence in THP1 (Mφ) cells incubated with A549‐derived exosomes of each group. FITC‐ Phalloidin (green) and Cy3 (red) were adopted for labeling cytoskeleton and miR‐19b‐3p, respectively. The percentages of Cy3‐positive macrophages were measured after certain hours of co‐culturing. Scale bar: 10 μm. (B) qRT‐PCR assessed miR‐19b‐3p level in A549 or H1975 cells transfected with pcDNA3.1 or pcDNA3.1/LINC00273. (C) qRT‐PCR assessed exosomal miR‐19b‐3p level from A549 or H1975 cells transfected with pcDNA3.1 or pcDNA3.1/LINC00273. (D) qRT‐PCR following RNA pull‐down assay evaluated the binding between miR‐19b‐3p and Bio‐LINC00273‐Sense or Antisense probe. (E) RBMX, the putative RBP for miR‐19b‐3p, was predicted by RBPDB. The nucleotides highlighted in green stand for the presumed binding site for RBMX in miR‐19b‐3p sequence or mutant binding site designed for RNA pull‐down assay. (F) Western blot following RNA pull‐down assay evaluated the enrichment of RBMX in the pulldown product of Bio‐NC, Bio‐miR‐19b‐3p‐WT or Bio‐miR‐19b‐3p‐Mut probe in LUAD cells or their exosomes. (G) RIP assay detected the relative input recovery of miR‐19b‐3p with anti‐RBMX or anti‐IgG in LUAD cells transfected with pcDNA3.1 or pcDNA3.1/LINC00273 or their exosomes. (H) qRT‐PCR assessed RBMX level in A549 or H1975 cells transfected with pcDNA3.1 or pcDNA3.1/LINC00273. (I) Western blot assessed exosomal RBMX and RBMX expression of LUAD cells transfected with pcDNA3.1 or pcDNA3.1/LINC00273. (J) Exosomes were extracted from A549 cells transfected with sh‐NC or sh‐RBMX#1. Fluorescence microscopy images illustrates the Cy3 fluorescence (red) in THP1 (Mφ) cells incubated with A549‐derived exosomes of each group. The percentages of Cy3‐positive macrophages were measured after certain hours. Scale bar: 10 μm. THP1 (Mφ), macrophages induced from THP1 cells. A549‐Exo or H1975‐Exo, exosomes derived from A549 or H1975 cells. Data are presented as mean ± SD of at least three independent experiments. *p < 0.05, **p < 0.01, ***p < 0.001, n.s., no significance

Since previously we found that M2 macrophages induced miR‐19b‐3p in LUAD cell‐derived exosomes rather than in LUAD cells, we explored whether M2 macrophage‐derived exosomal LINC00273 and its downstream Hippo/YAP pathway modulated the packaging of miR‐19b‐3p into LUAD cell‐derived exosomes. A549 cells were transfected with pcDNA3.1/LINC00273 and Cy3‐miR‐19b‐3p, and then the exosomes from the transfected A549 cells were added to the medium of THP1 (Mφ) cells for incubation. Consequently, the Cy3‐miR‐19b‐3p fluorescence was shown in THP1 (Mφ) cells incubated with A549‐derived exosomes, and the Cy3 fluorescence intensity was further enhanced in THP1 (Mφ) cells cultured with A549 exosomes with LINC00273 overexpression (Figure 8A and Supporting information Figure S5A). MiR‐19b‐3p expression was not changed under LINC00273 overexpression in LUAD cells (Figure 8B), excluding the modulatory effect of LINC00273 on miR‐19b‐3p expression in LUAD cells. However, we uncovered that miR‐19b‐3p was upregulated in LUAD cell‐derived exosomes after overexpressing LINC00273 (Figure 8C). RNA pulldown assay indicated that LINC00273 could not interact with miR‐19b‐3p (Figure 8D). Based on these data, we hypothesized that LINC00273 might promote the packaging of miR‐19b‐3p into LUAD cell‐derived exosomes.

Recent reports demonstrated that certain RBP may regulate the packaging of exosomal miRNAs such heterogeneous nuclear ribonucleoproteins (hnRNP A).^61^ Since we proved that LINC00273 regulated Hippo/YAP pathway in LUAD cells, we explored whether LINC00273 regulated miR‐19b‐3p packaging depending on certain factor in a Hippo/YAP‐dependent manner. We predicted the potential RBP matching the mature sequence of miR‐19b‐3p using the online prediction website RBPBD (threshold > 0.8). The results showed that RBMX was the only potential binding protein to miR‐19b‐3p (Figure 8E). RBMX is also a member of hnRNPs.^62^ Notably, RBMX is proved to interact with ARTS‐1 to facilitate the constitutive release of TNFR1 exosome‐like vesicles.^63^ Therefore, we hypothesized that RBMX helped the packaging of miR‐19b‐3p into LUAD cell‐derived exosomes. RNA pulldown plus western blot analysis confirmed that miR‐19b‐3p could bind to RBMX in LUAD cells, and the binding was more prevalent in LUAD cell‐derived exosomes than in LUAD cells (Figure 8F). RIP assay further demonstrated that overexpressing LINC00273 increased the binding between RBMX and miR‐19b‐3p in LUAD cells and exosomes (Figure 8G). In addition, overexpression of LINC00273 upregulated RBMX mRNA expression and increased both RBMX expression of LUAD cells and exosomal RBMX proteins (Figure 8H and I). Next, we knocked down RBMX in LUAD cells and confirmed the consequential downregulation of RBMX level in LUAD‐exosomes (Supporting information Figure S5B‐C). After THP1 (Mφ) cells were incubated with A549‐derived exosomes containing Cy3‐miR‐19b‐3p, the Cy3 fluorescence was observed in THP1 (Mφ) cells, and RBMX knockdown led to the decrease of Cy3 fluorescence in THP1 (Mφ) cells, indicating that RBMX knockdown reduced the transportation of exosomal miR‐19b‐3p from LUAD cells to THP1 (Mφ) cells (Figure 8J and Supporting information Figure S5D).

MiR‐19b‐3p expression was not altered when LUAD cells were cocultured with THP1 (Mφ) cell‐derived exosome with LINC00273 knockdown (THP1 (Mφ)/sh‐LINC00273#1) compared with THP1 (Mφ)/sh‐NC group (Supporting information Figure S5E). However, LUAD cell‐derived exosomal miR‐19b‐3p was upregulated when overexpressing LINC00273 in LUAD cells (Supporting information Figure S5F). Additionally, RBMX were downregulated in LUAD cells of THP1 (Mφ)/sh‐LINC00273#1 coculture group versus THP1 (Mφ)/sh‐NC coculture group (Supporting information Figure S5G‐H). Promoter transcription of RBMX in LUAD cells was probed by detecting the luciferase activity of RBMX‐Pro‐WT reporter. The luciferase activity was impeded in LUAD cells of THP1 (Mφ)/sh‐LINC00273#1 coculture group (Supporting information Figure S5I). In addition, the enhanced luciferase activity in LUAD cells transfected with LINC00273 expression vector (Supporting information Figure S5J). Furthermore, we detected the enhanced luciferase activity in LUAD cells treated with XMU‐MP‐1 (selective MST1/2 inhibitor) but the decreased activity in LUAD cells treated with Verteporfin (Supporting information Figure S5K). Thus, the knockdown of macrophage‐derived exosomal LINC00273 attenuated RBMX transcription and expression through Hippo/YAP pathway so as to inhibit the packaging of miR‐19b‐3p into LUAD cell‐derived exosomes.

Subsequently, we probed how LINC00273 regulated RBMX transcription through Hippo/YAP pathway in LUAD cells. We checked whether the transcription activator YAP affected RBMX transcription. Through ChIP assay, we determined that RBMX promoter was enriched in the immunoprecipitates conjugated with anti‐TEAD4 (Supporting information Figure S6A), indicating that TEAD4, the transcription factor activated by YAP in Hippo pathway, bound to RBMX promoter. Then, we overexpressed YAP in LUAD cells and detected the upregulation of YAP and RBMX (Supporting information Figure S6B‐C). Moreover, we uncovered that the enrichment of YAP in RBMX promoter was increased by LINC00273 overexpression but was weakened by Verteporfin (Supporting information Figure S6D). Luciferase reporter assay indicated that TEAD4 bound to RBMX promoter at several regions (Supporting information Figure S6E). Subsequently, the DNA motif of TEAD4 and its binding sequences in LINC00273 promoter were predicted using JASPAR and UCSC (Supporting information Figure S6F).

Further, by examining the luciferase activity of RBMX promoter reporter, we found that inhibiting YAP signaling by Veterporfin abolished RBMX promoter transcription at both site1 and site2 in LUAD cells with LINC00273 overexpression (Supporting information Figure S6G). Overexpressing YAP in LUAD cells activated RBMX promoter transcription at both site1 and site2 (Supporting information Figure S6H). Therefore, we determined that LINC00273 regulated RBMX transactivation through YAP and downstream TEAD4.

## 4 DISCUSSION

As revealed by former reports, tumor metastasis can be influenced by nonmalignant cells in the tumor microenvironment.^64^ Further, it has been proved that the nonmalignant macrophages in the tumor microenvironment can aggravate malignant progression through facilitating angiogenesis, promoting cancer cell migration and invasion, and restraining antitumor immunity.^65^ These reports supported the findings in our study that LUAD cells cocultured with macrophages were easier to metastasize to liver, lungs, and osseous tissues. However, macrophages are mainly polarized into M1‐ or M2‐like phenotypes, and their functions vary from one type to another.^12^ In tumor microenvironment, M1 macrophages usually elicit a tumor‐inhibiting function whereas M2 macrophages exert a tumor‐promoting function.^66^ Importantly, the findings that coculturing with LUAD cells led THP1 macrophages to malignancy‐promoting M2 polarization were revealed by Guo et al.^67^ These reports indicate that in LUAD microenvironment, inducing M2 polarization can facilitate tumor metastasis. Consistently, we validated that A549 cells cocultured with THP1 (Mφ) cells showed stronger metastasis in mice models. This result not only confirmed the participation of macrophages in LUAD metastasis, but also inferred that the macrophages might also be educated by LUAD cells to exert functions on LUAD cells in return, so we started with hypothesizing and investigating the influence of LUAD cells on M2 polarization.

We discovered that exosomes, the pivotal mediators of communication in the tumor microenvironment,^19^ were transported from LUAD cells to macrophages and induced M2 polarization. Previous reports supported that phosphorylated STAT3 induces macrophage polarization to M2‐like phenotype.^68^ Herein, we provided evidence that LUAD cell‐derived exosomes facilitated STAT3 phosphorylation in THP1 (Mφ) cells, leading to M2 polarization. Previously, PTPs have been suggested to induce STAT3 dephosphorylation and activation.^69^ PTPRD is a PTP member, and existing reports have proved that loss of PTPRD confers STAT3 activation in cancers like glioma^70^ and head and neck carcinoma.^71^ In our work, we identified that exosomes derived from LUAD cells reduced the expression of PTPRD to activate STAT3, resulting in M2 polarization of THP1 (Mφ) cells.

PTPRD is a protein‐coding gene whose expression can be regulated at diverse levels. Besides, over the past decades, miRNAs have been well‐proposed to silence protein‐coding genes at post‐transcriptional level.^72^ In present research, we screened out miR‐19b‐3p that was delivered by exosomes from LUAD cells into THP1 (Mφ) cells to target PTPRD. In addition, existing data have shown the upregulation of miR‐19b‐3p in the plasma of LUAD patients,^73^ supporting the participation of miR‐19b‐3p in LUAD. Our data illustrated that miR‐19b‐3p from LUAD cell derived‐exosomes contributed to M2 polarization of macrophages by targeting PTPRD. Interestingly, previous findings indicated that miR‐19b‐3p from exosomes of tubular epithelial cells results in M1 macrophage activation.^32^, ^74^ The contradiction between the reported results and our data indicated that the varying functions of miR‐19b‐3p from different cells might depend on tissue specificity. Subsequent data proved that miR‐19b‐3p overexpression‐induced M2 macrophages accelerated LUAD cell migration and invasion in vitro, and coculture of LUAD cells with miR‐19b‐3p‐silenced macrophages hindered tumor metastasis in vivo. In other words, miR‐19b‐3p‐induced M2 macrophages aggravated LUAD metastasis, which was consistent with the well‐accepted discovery that M2 macrophages exert oncogenic functions in cancers including LC.^16^, ^75^ Besides, numerous data presented the feedback regulatory loop where exosomes from cancer cells polarized macrophages to M2 phenotype, which reciprocally, aggravates malignant behaviors of cancer cells. For instance, Wang et al. supported that exosomal miR‐301a from tumor cells induces M2 macrophage polarization to accelerate metastasis in pancreatic cancer.^76^

Intriguingly, our study further found that inhibiting exosome secretion from M2 macrophages offset the facilitating effect of M2 macrophages on LUAD cell migration and invasion, suggesting M2 macrophages released exosomes to affect LUAD cell functions in return. In addition, we found that the transfer of exosomes from miR‐19b‐3p‐induced M2 macrophages into LUAD cells was more significant than that from THP1 (Mφ) cells to LUAD cells. This indicated the possibility that miR‐19b‐3p might influence the secretion of exosomes from M2 macrophages. However, we speculated that it was the molecules packaged in exosomes from M2 macrophages that actually went into LUAD cells and affected LUAD cells. Thus, we continued to focus on the molecular mechanism whereby M2 macrophage‐derived exosome influenced LUAD cells, and whether miR‐19b‐3p modulated exosome secretion from M2 macrophages will be explored in the future. Significantly, we discovered that exosomal miR‐19b‐3p from LUAD cells was regulated by YAP, the core protein downstream of LATS2 in Hippo pathway. In this pathway, phosphorylated LATS2 induces YAP phosphorylation, while only the unphosphorylated YAP could translocate into nucleus and activate gene transcription.^77^ Presently, we unmasked that miR‐19b‐3p‐induced M2 macrophage released exosomes to promote LATS2 ubiquitination and activate YAP in LUAD cells.

Protein ubiquitination is a multistep process modulated by proteins belonging to E1, E2, and E3 enzymes.^78^ LncRNAs, such as HOTAIR,^79^ are often suggested to function as a scaffold in regulating protein ubiquitination. In current work, LINC00273 was recognized as an exosomal lncRNA transferred from M2 macrophages to LUAD cells, affecting LATS2 ubiquitination by serving as the scaffold of NEDD4. NEDD4 is a HECT‐type E3 ligases which induces ubiquitination of tumor suppressive proteins to elicit oncogenic functions in diverse cancers.^80^, ^81^ Besides, the promoting role of NEDD4 in LC has been revealed by Shao et al.^82^ Meanwhile, we validated that LINC00273 expedited tumor metastasis in LUAD, which was concordant with its role previously found in many cancer types including LC.^29^, ^83^

In the end, we proved that LINC00273 increased miR‐19b‐3p in the exosomes of LUAD cells by elevating YAP‐activated RBMX. Originally, RBMX was identified as a nuclear RNA‐binding protein that belongs to hnRNPs.^62^ Nonetheless, there is also a former study demonstrating that RBMX could interact with ARTS‐1 to accelerate the constitutive release of TNFR1 exosome‐like vesicles.^63^ Similarly, our findings verified that RBMX interacted with miR‐19b‐3p to facilitate the packaging of miR‐19b‐3p into exosomes, which might be explained by the involvement of hnRNPs in the sorting of miRNAs into exosomes.^84^

## 5 CONCLUSIONS

**FIGURE 9:**
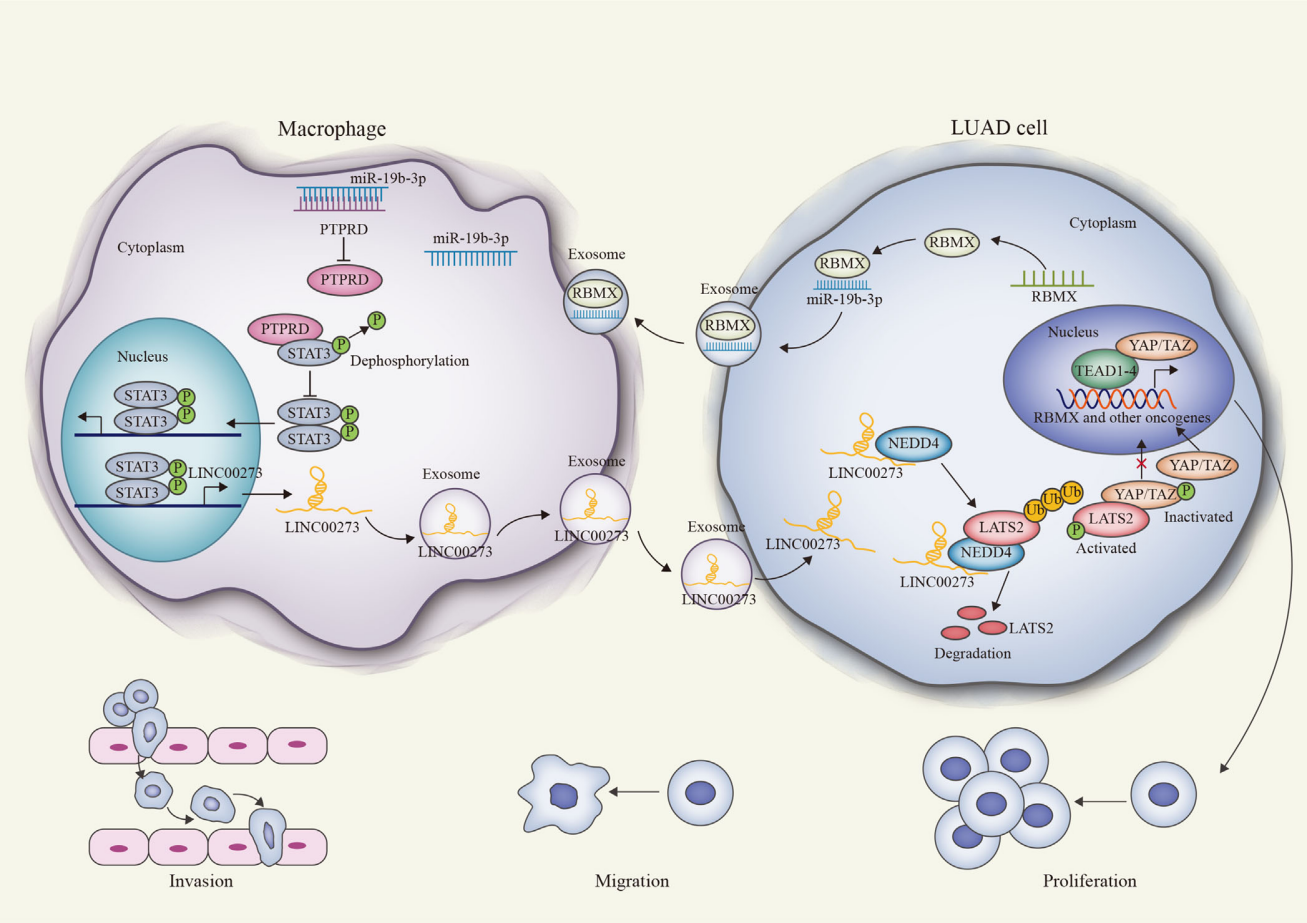
Schematic diagram of tumor‐derived exosomal miR‐19b‐3p facilitates M2 macrophage polarization and exosomal LINC00273 secretion to promote lung adenocarcinoma metastasis via Hippo pathway

In conclusion, our work elucidated the crosstalking between TAMs and LUAD cells mediated by exosomes (Figure 9). In detail, exosomes from LUAD cells deliver miR‐19b‐3p to macrophages to target PTPRD and activate STAT3 signaling, and the activated STAT3 induces M2 macrophage polarization and LINC00273 transcription. In return, M2 macrophages transmit exosomal LINC00273 to LUAD cells to induce LATS2 ubiquitination and activate YAP, so that the activated YAP transcriptionally activates RBMX to facilitate the miR‐19b‐3p packaging into exosomes from LUAD cells. Our findings might provide new possibilities for LUAD treatment development.

## CONFLICT OF INTEREST

The authors declare that they have no conflict of interest.

## Supporting information

SUPPORTING INFORMATION

SUPPORTING INFORMATION

SUPPORTING INFORMATION

SUPPORTING INFORMATION

SUPPORTING INFORMATION

SUPPORTING INFORMATION

SUPPORTING INFORMATION

SUPPORTING INFORMATION

SUPPORTING INFORMATION

SUPPORTING INFORMATION

SUPPORTING INFORMATION

## Data Availability

Not applicable.
