# Supplementary figures and images for: Tumor‐derived exosomal miR‐19b‐3p facilitates M2 macrophage polarization and exosomal LINC00273 secretion to promote lung adenocarcinoma metastasis via Hippo pathway
Source: Clin Transl Med. 2021 Sep 12;11(9):e478. doi: 10.1002/ctm2.478 (PMC8435259; doi:10.1002/ctm2.478)

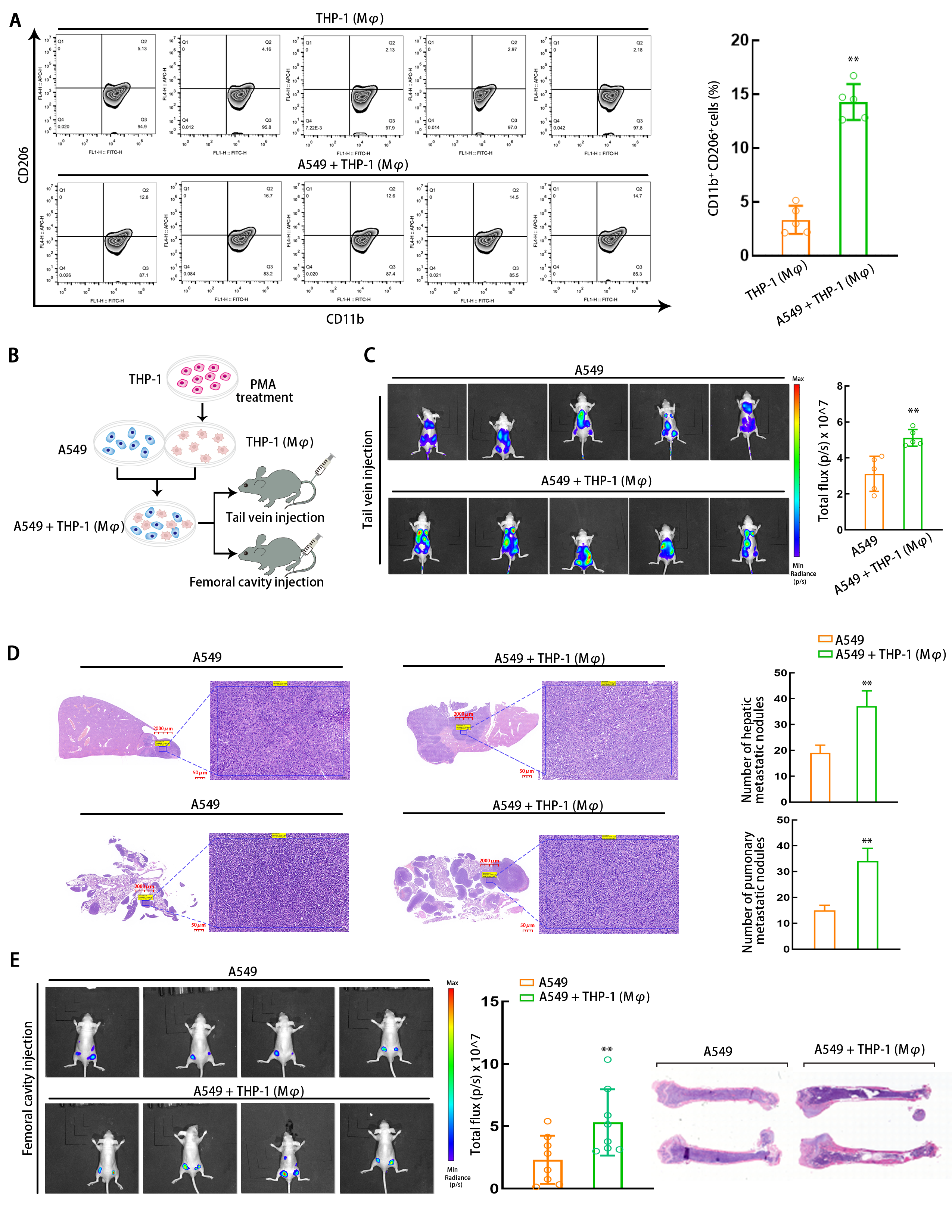

Supplement: Supplementary file 3 — SUPPORTING INFORMATION [file CTM2-11-e478-s002.tif]

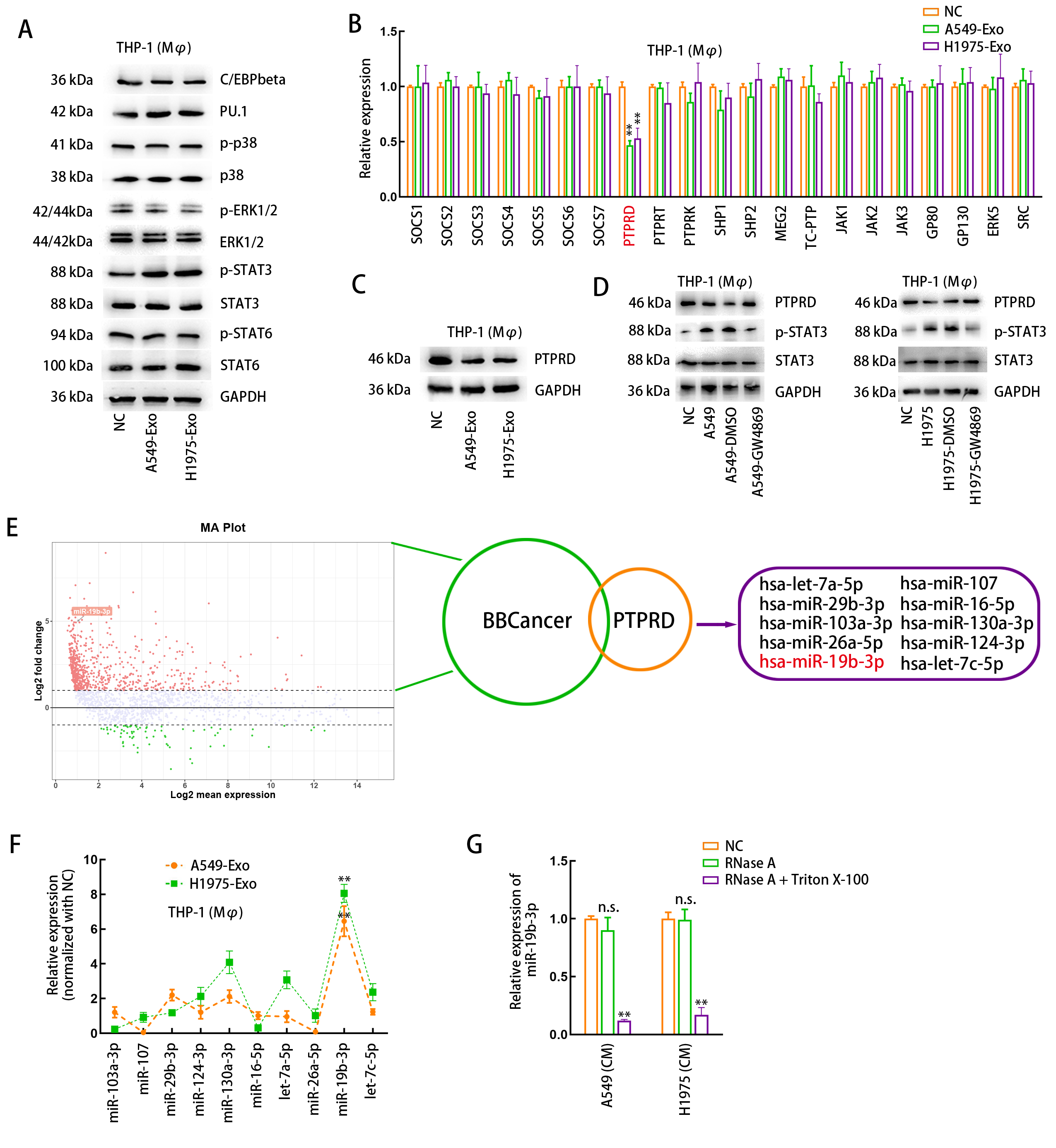

Supplement: Supplementary file 4 — SUPPORTING INFORMATION [file CTM2-11-e478-s001.tif]

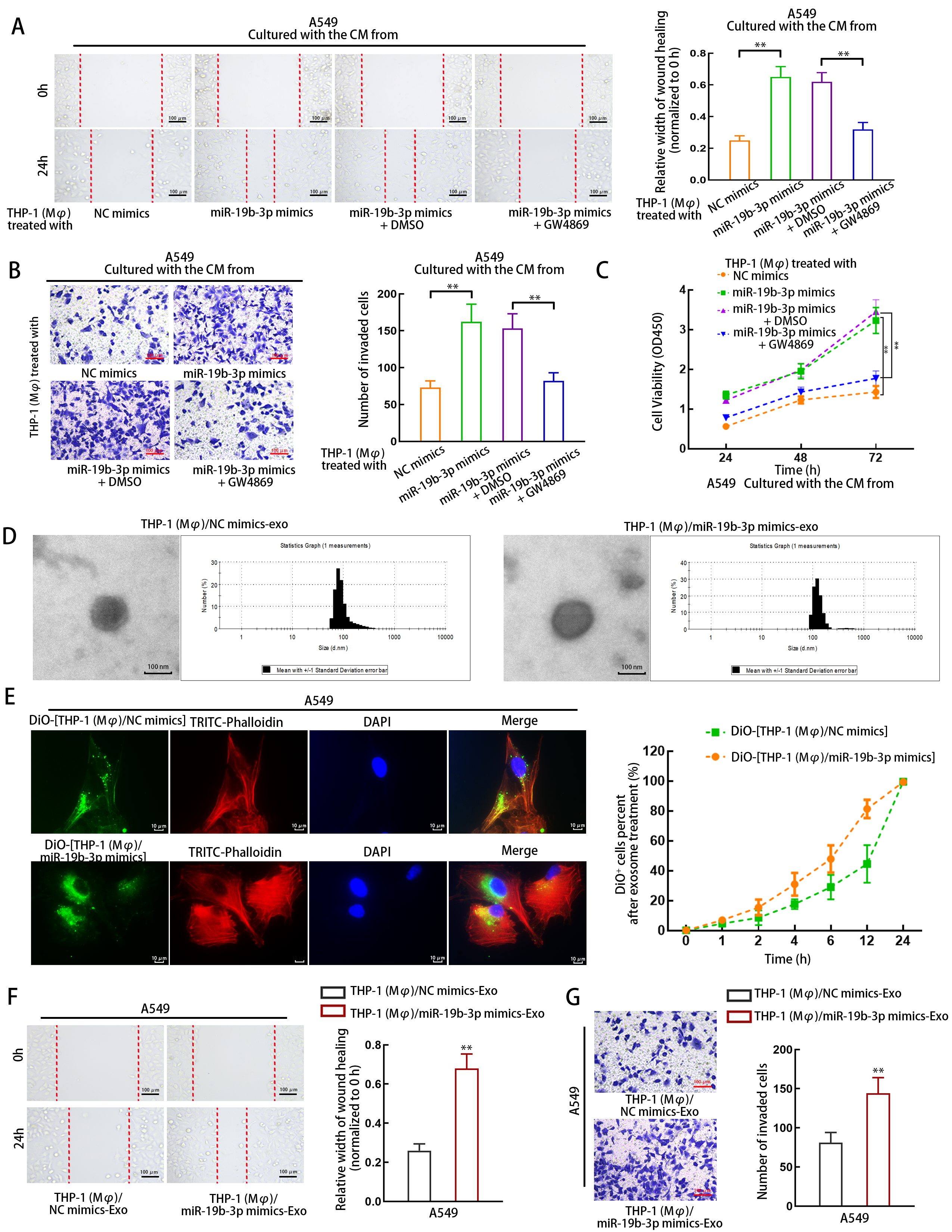

Supplement: Supplementary file 5 — SUPPORTING INFORMATION [file CTM2-11-e478-s008.tif]

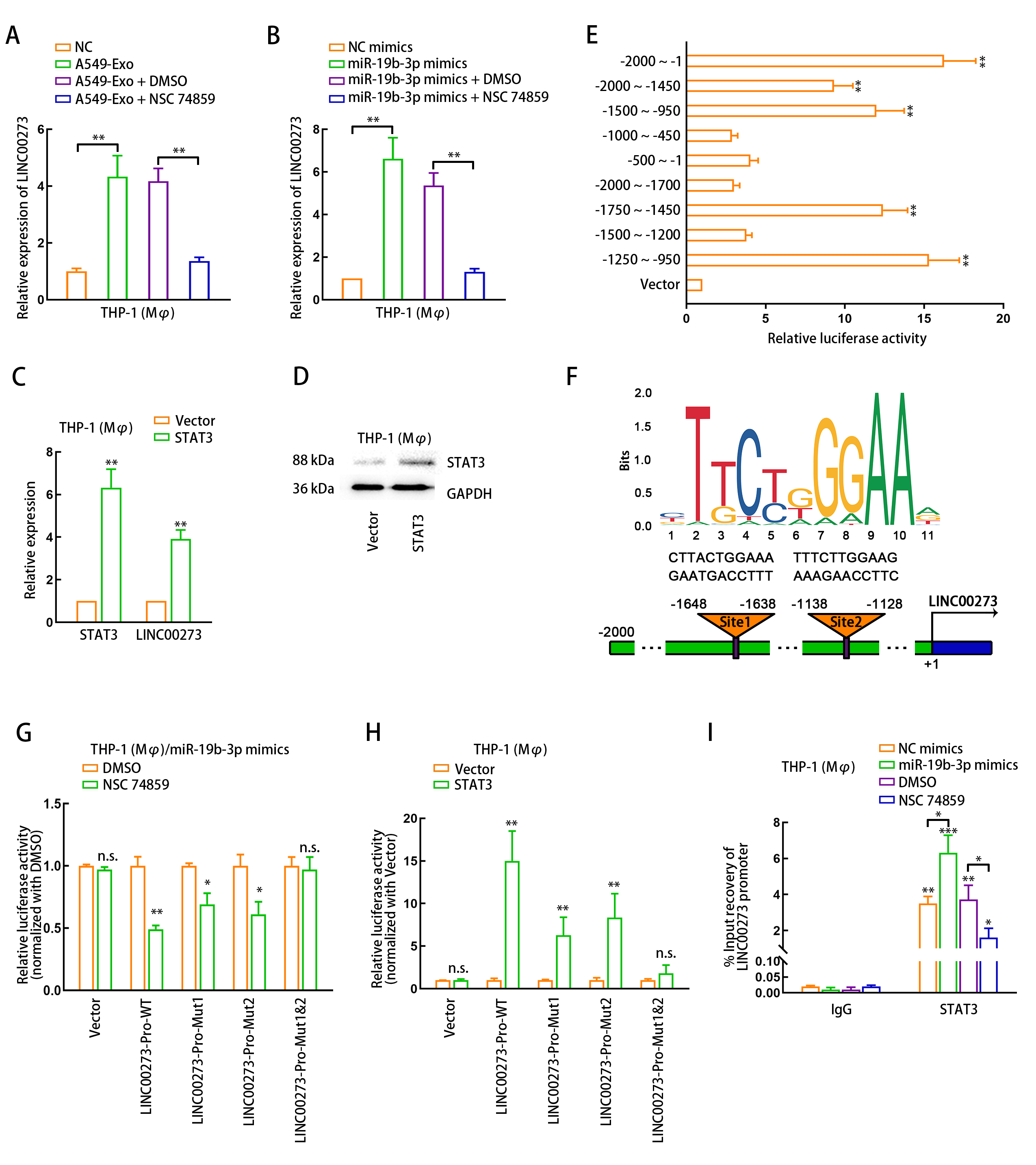

Supplement: Supplementary file 6 — SUPPORTING INFORMATION [file CTM2-11-e478-s009.tif]

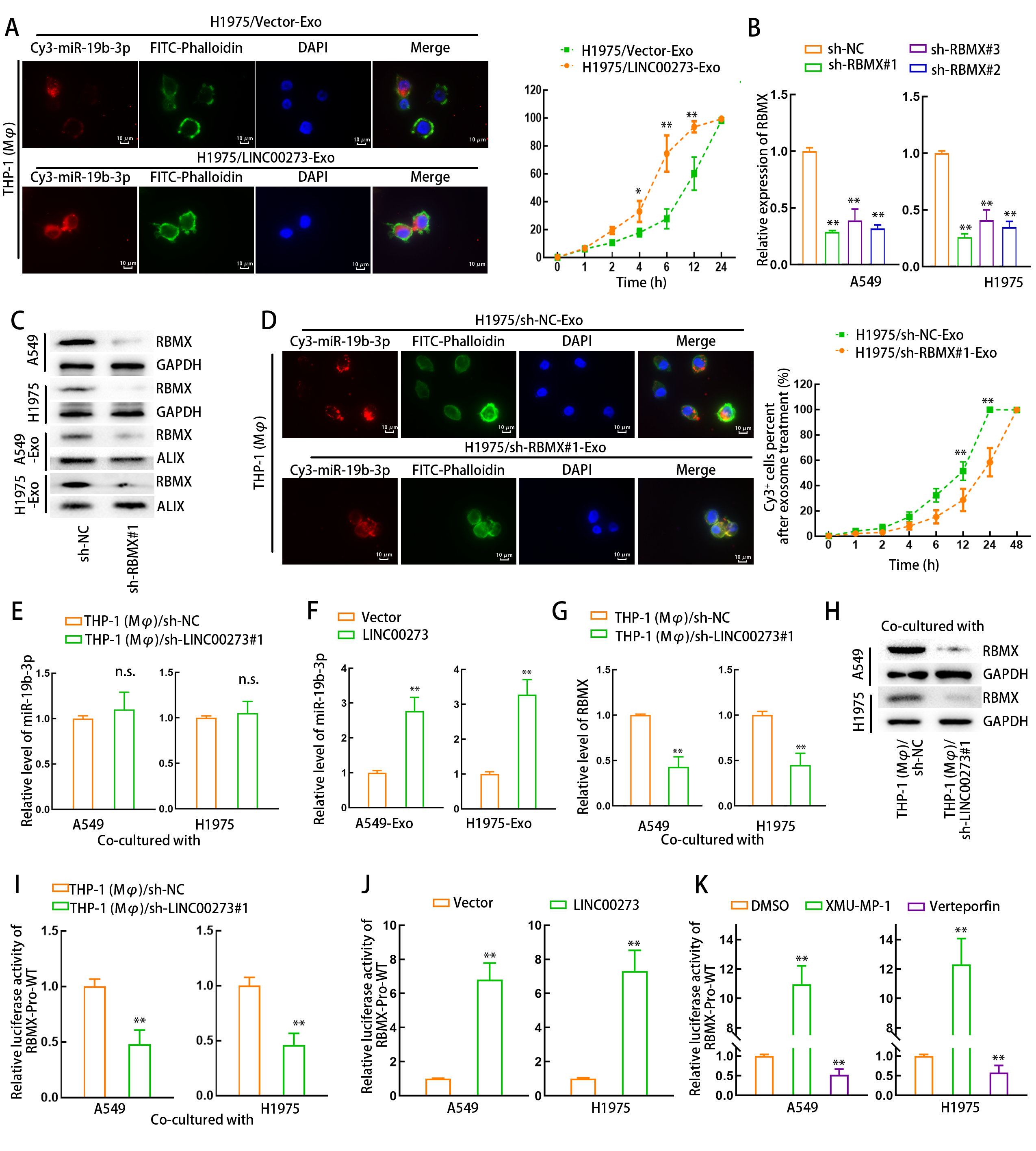

Supplement: Supplementary file 7 — SUPPORTING INFORMATION [file CTM2-11-e478-s006.tif]

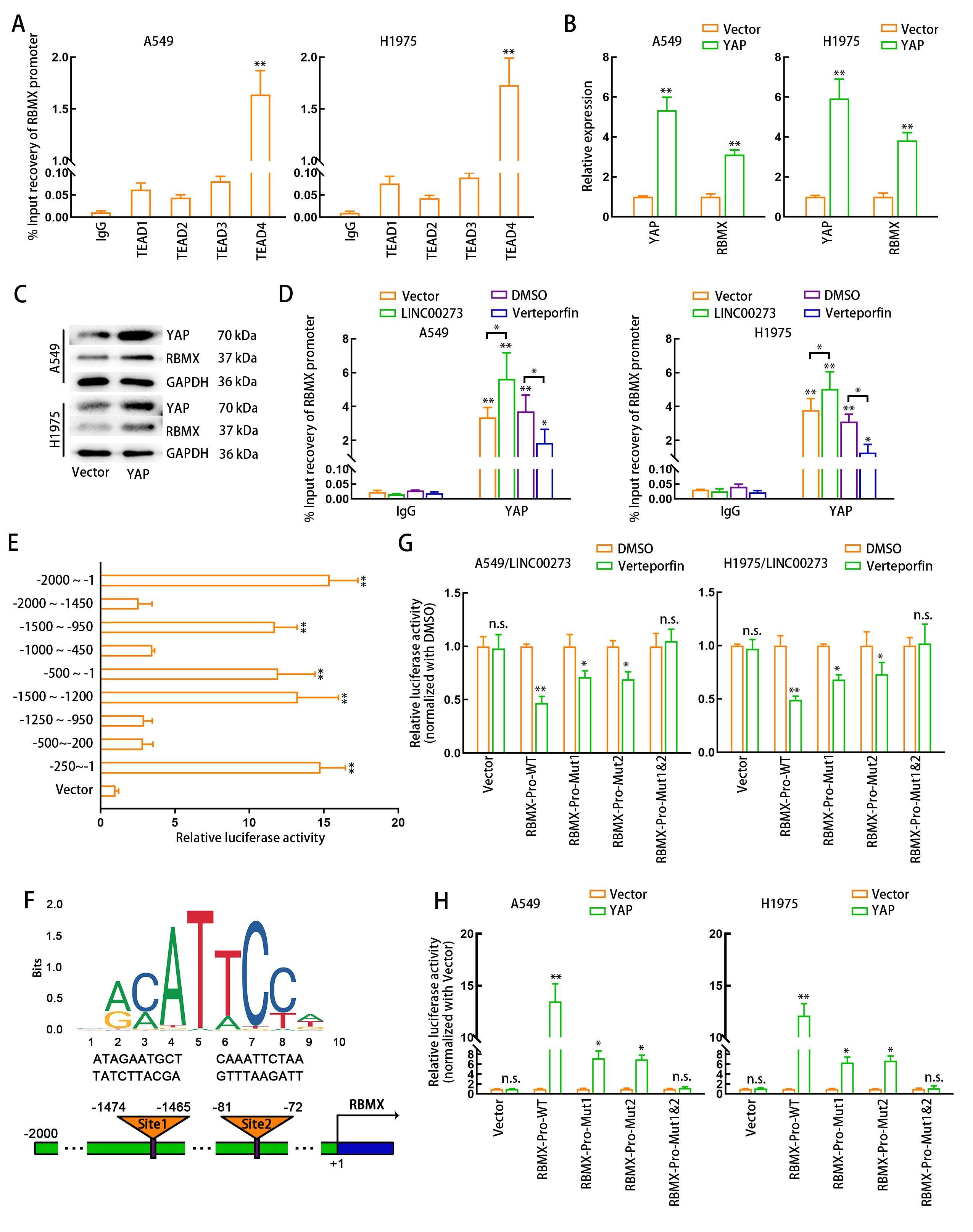

Supplement: Supplementary file 8 — SUPPORTING INFORMATION [file CTM2-11-e478-s007.tif]

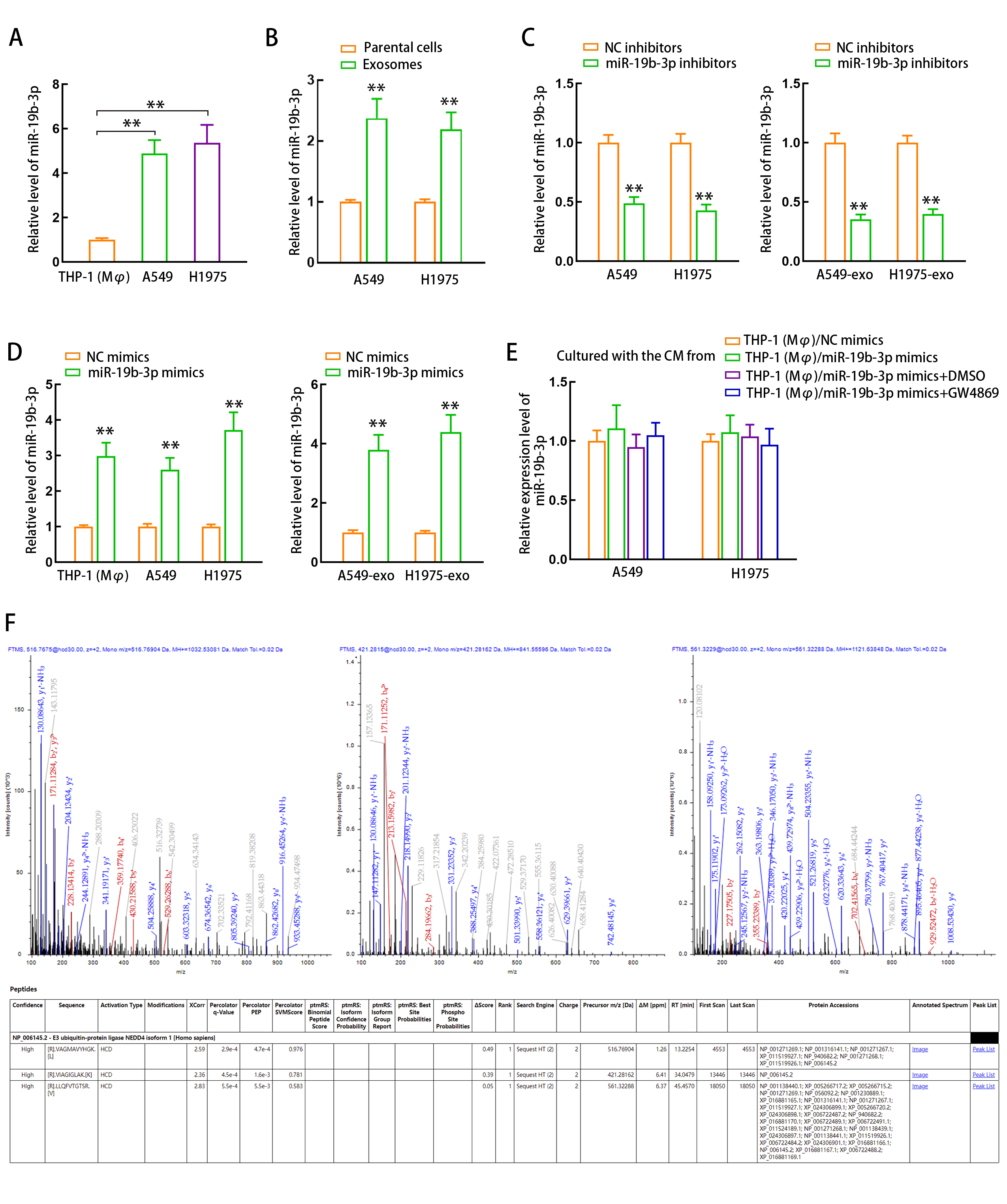

Supplement: Supplementary file 9 — SUPPORTING INFORMATION [file CTM2-11-e478-s010.tif]

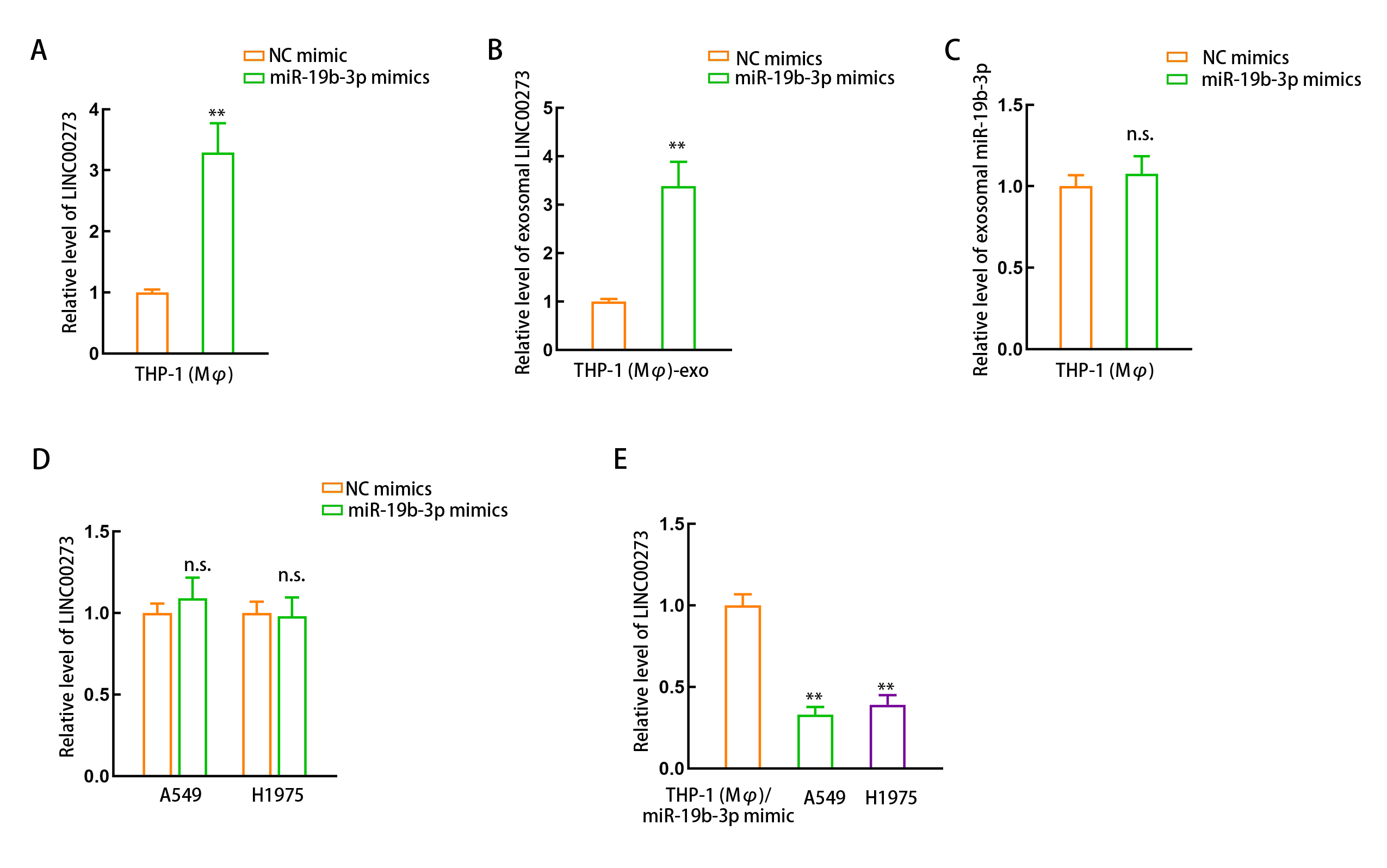

Supplement: Supplementary file 10 — SUPPORTING INFORMATION [file CTM2-11-e478-s005.tif]

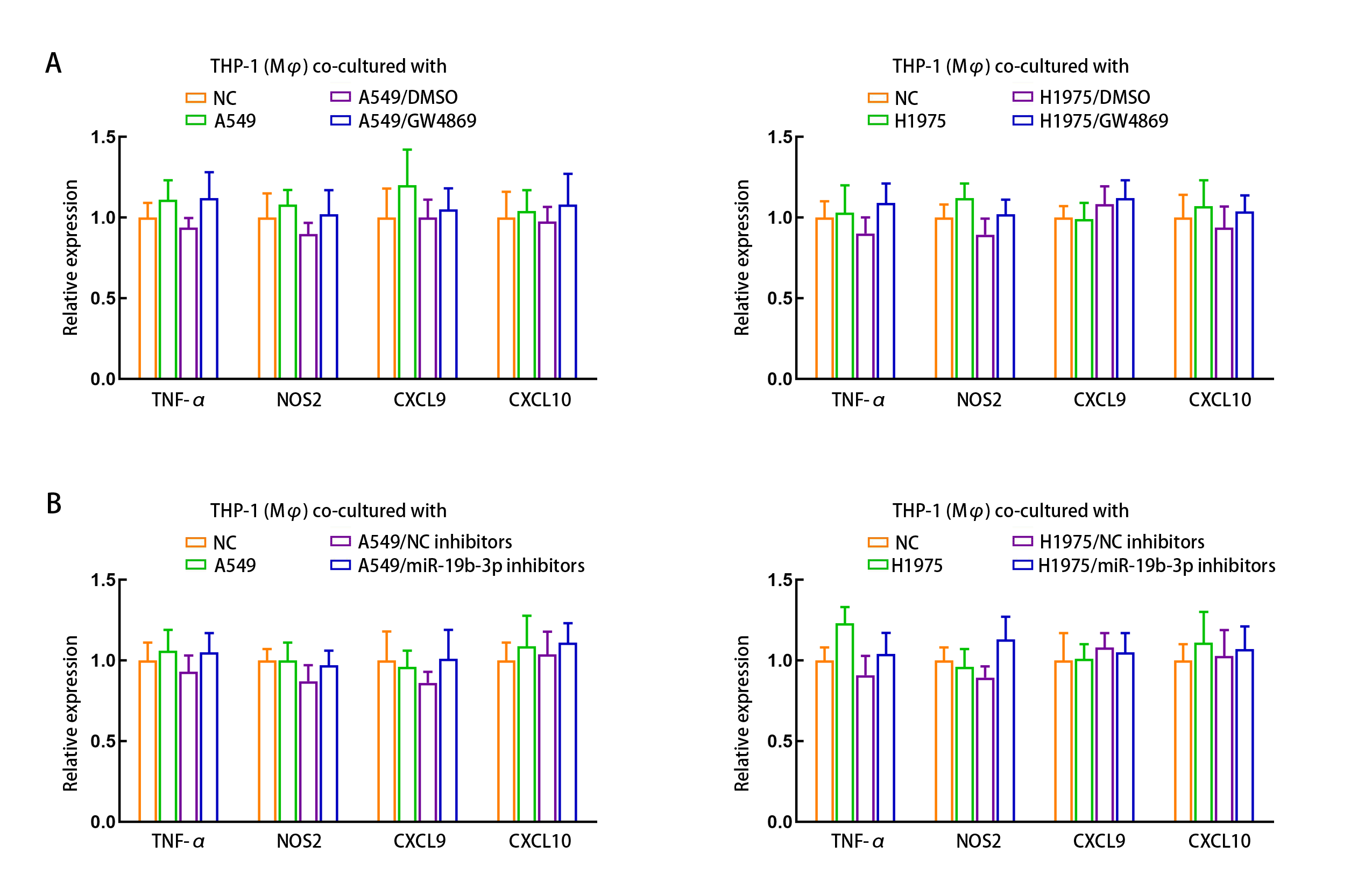

Supplement: Supplementary file 11 — SUPPORTING INFORMATION [file CTM2-11-e478-s011.tif]
